# Carrier-free self-assembled nanodrugs in cancer therapy: a systematic scoping review and evidence map of molecular architecture, drug resistance, and translation-relevant reporting

**DOI:** 10.3389/fphar.2026.1977190

**Published:** 2026-10-09

**Authors:** Yifei Wang, Lulan Ma, Jiangshan Hu, Yanqiu Liu, Bonian Zhao, Yan Gao

**Affiliations:** 1 Institute of Pharmaceutical Research, Shandong University of Traditional Chinese Medicine, Jinan, Shandong, China; 2 High-Level Traditional Chinese Medicine Key Discipline of the State Administration of Traditional Chinese Medicine Pharmaceutics of Traditional Chinese Medicine, Jinan, Shandong, China; 3 Collaborative Innovation Center for Ecological Protection and High-Quality Development of Characteristic Traditional Chinese Medicine in the Yellow River Basin, Jinan, Shandong, China; 4 Shandong Key Laboratory of Digital Traditional Chinese Medicine, Shandong University of Traditional Chinese Medicine, Jinan, Shandong, China; 5 Shandong University of Traditional Chinese Medicine Yusen Pharmaceutical Technology Co., Ltd., Jinan, Shandong, China; 6 Innovative Institute of Chinese Medicine and Pharmacy, Shandong University of Traditional Chinese Medicine, Jinan, Shandong, China

**Keywords:** bibliometric analysis, cancer nanomedicine, carrier-free nanodrugs, drug resistance, evidence mapping, self-assembly, systematic review, translation-relevant reporting

## Abstract

**Background:**

Carrier-free self-assembled nanodrugs use therapeutic or bioactive molecules as principal structural components. Their architectures have diversified rapidly, but how molecular design relates to resistance evidence and further development remains unclear.

**Objective:**

To map molecular architecture, drug-resistance evidence, and translation-relevant reporting in carrier-free anticancer nanodrugs.

**Methods:**

We conducted a PRISMA-ScR systematic scoping review and evidence map integrated with bibliometric analysis and structured appraisal of key resistance studies. PubMed, Europe PMC, Scopus, and Web of Science Core Collection (WoSCC) were searched in August 2026. WoSCC records (n = 863) characterized publication and intellectual structure, Scopus records (n = 840) assessed coverage, and 526 eligible articles formed the evidence map. Architecture was classified in 481 original studies, resistance evidence in 160 records, and 19 translation-relevant domains in 147 full-text studies. All 43 Tier 1/2 studies underwent full-report appraisal.

**Results:**

Covalent prodrug/conjugate assemblies (n = 187) and noncovalent multi-active co-assemblies (n = 182) predominated. Of 160 resistance-related records, 28 used established resistant models (Tier 1), whereas 15 provided mechanism-aligned sensitization evidence without resistant-model confirmation (Tier 2). Architecture was not clearly associated with Tier one versus Tier 2 classification in exploratory analysis (Cramér’s V = 0.394, Holm-adjusted P = 0.666). In the 147-study full-text subset, batch consistency and experimental scalability were each reported in three studies and long-term toxicity in four. Seven studies met the composite human-related evidence domain, but only one involved direct clinical administration (1/147, 0.7%). After excluding incomplete 2026 data, the latest-versus-earliest difference in reporting completeness was 1.40 domains (95% CI, −0.49–3.25), providing no clear evidence of sustained temporal improvement.

**Conclusion:**

Carrier-free anticancer nanodrugs have diversified rapidly, but evidence supporting resistance and further development has not matured in parallel. By separating resistance mechanism from evidentiary strength and distinguishing resistant-model validation from mechanism-aligned sensitization, this review establishes clearer boundaries for resistance claims and identifies manufacturing reproducibility, long-term safety, and direct clinical evaluation as major evidence gaps. The resulting framework provides a practical basis for aligning molecular design with claim-specific pharmacology and development-oriented evidence generation.

## Introduction

Nanomedicine can improve drug formulation, systemic exposure, and tumor delivery, but conventional nanocarriers also introduce inactive materials, additional quality attributes, and uncertainties related to degradation and clearance (Wilhelm et al., 2016; Hare et al., 2017; Shi et al., 2017; Mitchell et al., 2021). These limitations have encouraged carrier-free systems in which therapeutic or bioactive molecules form the principal structural components of nanoscale formulations (Yang et al., 2019; Karaosmanoglu et al., 2021). Carrier-free nanodrugs now span pure-drug particles, covalent prodrug/conjugate assemblies, noncovalent multi-active co-assemblies, metal-coordinated systems, and biomacromolecular assemblies (Zhang X. et al., 2023; An et al., 2024; Ji et al., 2024). Many also integrate drug delivery with imaging, phototherapy, immune modulation, and other therapeutic functions (Zhang et al., 2015; Liang et al., 2017). This diversification raises an important pharmacological question: whether increasingly sophisticated molecular architectures are accompanied by evidence linking material design to drug exposure, treatment response, and further development (Bhatia et al., 2022; Fang and Chen, 2024).

Carrier-free status does not, however, imply a uniform material state or simplified pharmaceutical development. Pure-drug particles remain sensitive to crystallinity, aggregation, and dissolution, whereas covalent prodrug assemblies depend on conjugation, chemical purity, linker stability, and controlled cleavage (Huang et al., 2014; Shim et al., 2019). Noncovalent co-assemblies are governed by intermolecular interactions and may be sensitive to dilution and biological media (Zhang et al., 2016; Li et al., 2017), while coordination-based and biomacromolecular systems introduce additional dependence on metal-ligand interactions, molecular composition, and biological stability (Fan et al., 2018; Wang et al., 2021). Accessory ligands, membranes, and stabilizing layers can further alter colloidal behavior and biological disposition (Ji et al., 2018; Zhang et al., 2018). Carrier elimination therefore shifts rather than removes formulation complexity, and changes in assembly state, stability, release, or biomolecular interactions can directly alter pharmacological exposure (Monopoli et al., 2012).

Drug resistance provides a particularly useful setting in which molecular design can be distinguished from the evidence supporting a pharmacological claim. Some carrier-free systems have increased intracellular drug retention, inhibited efflux, and restored sensitivity in established resistant cancer models (Wang et al., 2014; Zhao et al., 2015; Xiao et al., 2018; Gao et al., 2020). Others have targeted epigenetic regulation, autophagy, or stem-like phenotypes to enhance treatment sensitivity (Xu et al., 2017; Xin et al., 2019; Xie et al., 2022), while broader resistance-related strategies have addressed hypoxia, ferroptosis defense, and immune suppression (Zhu et al., 2019; Long et al., 2020). These mechanisms are all pharmacologically relevant, but they do not support equivalent claims. Resistant-model validation should therefore be distinguished from mechanism-aligned sensitization and from other treatment-resistance contexts. We selected resistance as the principal evidentiary anchor because increased uptake, intracellular retention, or cytotoxicity may reflect altered exposure without demonstrating reversal of an established resistant phenotype. Interpreting the model, comparator, mechanism, and pharmacological endpoint together provides a more discriminating assessment than counting efficacy or pathway-related endpoints alone.

Existing reviews have described carrier-free design principles, assembly mechanisms, therapeutic applications, and translational challenges (Yang et al., 2019; Karaosmanoglu et al., 2021; Zhang X. et al., 2023; An et al., 2024; Fang and Chen, 2024; Ji et al., 2024), while bibliometric studies have mapped the growth and thematic evolution of cancer nanomedicine (Teles et al., 2018; Zhu X. et al., 2020; Pei et al., 2022; Sun et al., 2023; Chen et al., 2024; Tao et al., 2024; Zhang et al., 2025), including nanomedicine and drug resistance (Wang et al., 2022; Duan et al., 2025). However, bibliometric trends do not establish the architecture or pharmacological evidence of individual nanosystems, and conventional reviews rarely quantify development-relevant reporting across the literature. In our prespecified comparison, we identified no previous review combining a carrier-free scope with article-level molecular-architecture classification, claim-oriented resistance assessment, and systematic evaluation of translation-relevant reporting (Supplementary Table S1).

We therefore conducted a systematic scoping review and evidence map integrated with bibliometric analysis to characterize the molecular architectures and therapeutic functions of carrier-free anticancer nanodrugs, evaluate the experimental evidence supporting drug-resistance claims, and assess translation-relevant reporting. Original studies were classified by primary molecular architecture, resistance evidence was assigned to four claim-oriented categories, and full-text studies were assessed across 19 domains covering formulation characterization, stability, pharmacokinetics, safety, manufacture, and human relevance. The resulting evidence map links molecular design with pharmacological claim strength and development-relevant evidence.

## Methods

### Study design and eligibility

We conducted a systematic scoping review and evidence map with bibliometric analysis to examine molecular architecture, drug-resistance evidence, and translation-relevant reporting in carrier-free anticancer nanodrugs. The review followed PRISMA-ScR, with relevant PRISMA 2020 items applied where appropriate (Tricco et al., 2018; Page et al., 2021). Bibliometric analyses followed established guidance (Donthu et al., 2021; Montazeri et al., 2023). The review was not prospectively registered.

Eligible records were English-language journal articles or reviews describing cancer-related nanoscale systems in which therapeutic or bioactive molecules formed the principal structural component. These included pure-drug particles, covalent prodrug/conjugate assemblies, noncovalent multi-active co-assemblies, metal-coordinated systems, biomacromolecular assemblies, and drug-dominant systems with accessory targeting or stabilizing components. Studies were excluded if they addressed non-cancer applications, lacked a nanoscale drug-dominant formulation, used ineligible publication types, or relied mainly on conventional inactive carriers such as lipids, polymers, protein matrices, silica, or metal-organic frameworks. Reviews were retained for bibliometric analysis but excluded from article-level evidence assessment. Detailed criteria are provided in Supplementary Table S2.

Carrier-free status was determined by structural composition rather than by the preparation method alone. Accessory PEG, hyaluronic acid, targeting ligands, or membranes were permitted when the therapeutic components remained the principal structural phase. Active peptides, proteins, or nucleic acids could also serve as structural components. Nanoprecipitation, antisolvent precipitation, microfluidic mixing, and crystallization were considered preparation routes and were not taken as evidence of a specific self-assembly mechanism.

### Literature search and data processing

A comprehensive literature search was conducted in PubMed, Europe PMC, Web of Science Core Collection (WoSCC), and Scopus to identify studies on carrier-free self-assembled nanodrugs in cancer therapy. The search strategy combined carrier-free identity terms with nanodrug and cancer-related concepts. Complete database-specific search strategies, field codes, filters, and retrieval results are provided in Supplementary Table S3. The WoSCC strategy was developed with reference to PubMed and Europe PMC and then adapted for Scopus. Retrieval sensitivity was checked against representative reviews and landmark studies, and a broader drug-drug co-assembly search was also tested. PubMed and Europe PMC were searched on 12 August 2026, Scopus on 16 August 2026, and WoSCC on 17 August 2026. The final searches yielded 863 WoSCC records and 840 Scopus records.

Records were normalized and deduplicated using database identifiers, DOI, and title. Cross-database matching used normalized DOI with exact normalized title as a fallback, linking 727 records between WoSCC and Scopus, while 136 WoSCC records and 113 Scopus records remained database-specific. The two databases were therefore analyzed separately, with Scopus used for coverage sensitivity. The evidence map was derived from PubMed and Europe PMC. After deduplication, 588 records were screened and 526 were eligible, including 481 original studies. Translation-relevant reporting was assessed in 147 available full-text original studies. Yifei Wang performed the complete initial title/abstract screening, eligibility assessment using available source information, data extraction, architecture classification, resistance classification, and translation-domain assessment. Jiangshan Hu independently assessed the specified samples and all 43 key resistance studies without access to the first evaluator’s decisions. Duplicate assessment covered these documented samples and the key resistance module, not the complete screening and extraction workflow. The two evaluators resolved disagreements by discussion after recording their independent decisions. The analytical datasets, denominators, and interpretation boundaries are summarized in Table 1.

**TABLE 1:** Analytical datasets, denominators, and interpretation boundaries. Summary of the source-specific datasets used for bibliometric analysis, database-coverage assessment, and article-level evidence mapping. Analytical denominators were retained separately, and data from different sources were not pooled.

Analytical layer	Source	Denominator	Primary analytical use	Permitted interpretation	Prohibited extrapolation
Search-defined bibliometric corpus	Web of Science Core Collection	863 records	Publication performance, source distribution, author keywords, bibliographic coupling, and reference co-citation	Bibliometric and intellectual structure of the WoSCC search-defined corpus	Not an article-level eligibility-adjudicated corpus; database counts are not pooled with Scopus
Coverage-sensitivity corpus	Scopus	840 records	Cross-database coverage comparison and DOI/title overlap	Sensitivity of retrieval coverage to database choice	Not merged with WoSCC citation counts or treated as a second independently screened evidence corpus
Eligibility-screened evidence map	PubMed and Europe PMC public-source workflow	526 eligible articles	Article-level eligibility review and evidence mapping	Evidence structure among the 526 eligible articles	Not interchangeable with the 863-record WoSCC corpus or the 840-record Scopus corpus
Original-study evidence layer	Original studies within the evidence map	481 original studies	Primary molecular-architecture classification and experimental evidence analyses	Architecture distribution among eligible original studies	Does not include the 45 reviews and does not imply full-text availability for every study
Full-text translation-relevant reporting assessment	Locally available full-text original-study subset	147 original studies	Nineteen-domain translation-relevant reporting assessment and reporting-completeness analyses	Reporting frequencies within the audited full-text subset	Must not be extrapolated as prevalence estimates for all 481 original studies or the wider field

Verification samples were selected by deterministic SHA-256 ordering within predefined groups: 40 included and 40 excluded screening records; 12 records from each of five principal architecture classes plus the single other/unclear record; and 10 records from each resistance category. For translation reporting, studies were ordered by score and assigned cyclically to four interleaved groups, with five selected per group (20 studies and 380 domain decisions). These groups were not contiguous score quartiles, and the samples were not simple random samples of the entire corpus. The 43-study key resistance module was assessed separately. Supplementary Table S12 provided the selection procedure and pre-consensus agreement estimates.

### Bibliometric analysis and evidence mapping

Bibliometric analyses used the complete WoSCC corpus to examine publication trends, source distribution, author keywords, bibliographic coupling, and reference co-citation. Keywords were normalized for case and punctuation without merging scientifically distinct terms. Temporal patterns were summarized for publications up to 2015, 2016-2020, and 2021-2026, with the final period treated as incomplete. Bibliographic coupling and co-citation networks were generated in bibliometrix 5.4.1 (Aria and Cuccurullo, 2017) following established approaches (van Eck and Waltman, 2010; Donthu et al., 2021). Weighted Louvain clustering was used to identify intellectual communities.

Article-level evidence mapping was performed on the screened original studies. Each of the 481 studies was assigned one primary molecular architecture: pure-drug, covalent prodrug/conjugate, noncovalent multi-active, metal-coordinated, biomacromolecular, or other/unclear. Accessory features, including PEGylation, targeting ligands, membranes, and stabilizing coatings, were recorded separately, and therapeutic modalities were coded non-exclusively. Rule-assisted preliminary classifications were manually checked in a prespecified 71-record sample, with agreement of 53/71 (74.6%). Because this was below the predefined threshold, all 481 original studies underwent manual adjudication. Operational definitions and classification rules are provided in Supplementary Table S4.

### Resistance evidence and translation assessment

Resistance-related studies were classified into four claim-oriented categories. Tier one required an established resistant model, or paired sensitive and resistant models, with an interpretable comparator. Tier 2 included mechanism-aligned resistance or sensitization evidence without resistant-model confirmation. Tier 3 covered other experimentally studied resistance contexts, including hypoxia-related photodynamic resistance, thermotolerance, immune escape, and ferroptosis defense. Tier 4 indicated resistance language without direct experimental evidence. These categories do not constitute a universal ranking of study quality, and resistance mechanisms were coded separately and non-exclusively (Supplementary Table S5). Accordingly, tier numbers should not be interpreted as a ranking of evidentiary rigor across different resistance-mechanism classes.

The 43 source-verified Tier one and Tier 2 studies underwent structured methodological appraisal. Because the studies included heterogeneous *in vitro*, animal, and mixed designs, no composite quality score was calculated. Appraisal covered resistance-model alignment, comparator reporting, formulation characterization, replication or sample-size reporting, statistical methods, and, where applicable, randomization and blinding. Study-level results are provided in Supplementary Table S14.

Translation-relevant reporting was assessed across 19 predefined domains in 147 full-text original studies (Supplementary Table S6). Domains covered formulation identity, physicochemical characterization, stability, efficacy, pharmacokinetics, safety, manufacturing, and human relevance, and were informed by MIRIBEL and published nanomedicine guidance (Crist et al., 2013; Tinkle et al., 2014; D’Mello et al., 2017; Faria et al., 2018; Halamoda-Kenzaoui et al., 2019; Leong et al., 2019; Germain et al., 2020; U.S. Food and Drug Administration, 2022). A domain was scored as present only when the report contained study-level evidence meeting its predefined criteria. For the human-related domain, eligible evidence included direct clinical administration, patient- or donor-derived experimental material, or patient-derived molecular datasets.

Automated phrase matching was used only to identify candidate evidence, followed by article-level confirmation. Prespecified negative samples were reviewed to identify possible false negatives. The final 147-study matrix was used to calculate reporting completeness, which reflects reporting breadth rather than formulation quality, efficacy, regulatory readiness, or clinical success. Differences between the full-text subset and the remaining original studies were assessed separately. Each domain contributed one point to the descriptive completeness score. This equal numerical contribution was used solely to summarize reporting breadth and should not be interpreted as indicating equal scientific, clinical, or regulatory importance across domains.

### Statistical analysis

Reporting frequencies in the 147-study full-text subset were summarized using counts, percentages, and Wilson 95% confidence intervals (Brown et al., 2001). Reporting-completeness scores were summarized using medians and interquartile ranges. Temporal comparisons were evaluated using mean differences with nonparametric bootstrap 95% confidence intervals based on 10000 resamples, with sensitivity analysis excluding the incomplete 2026 publication year. Domain-specific counts and confidence intervals are provided in Supplementary Table S9.

Differences between the full-text subset and the remaining original studies were evaluated descriptively using standardized differences for publication year and architecture-specific proportions, together with Cramér’s V for overall architecture distribution. Architecture-specific standardized differences used the pooled overall prevalence in the denominator, as reported in Supplementary Table S8. These measures describe availability-related differences rather than random-sampling representativeness. Independent duplicate classifications were assessed using raw agreement and unweighted Cohen’s kappa (Cohen, 1960). Because the four resistance categories do not form a universal quality ranking, unweighted kappa was the primary measure and no inference depended on ordinal distances between categories.

Exploratory association analyses examined architecture in relation to Tier one versus Tier 2 classification in the 43 key resistance studies and to pharmacokinetics, colloidal stability, serum stability, storage stability, and *in vivo* efficacy reporting in the 147-study subset. Sparse contingency tables were analyzed using two-sided Fisher tests with 100,000 Monte Carlo replicates. Cramér’s V described association magnitude, Wilson intervals were calculated for within-architecture proportions, and Holm correction was applied across six tests. Sensitivity analyses excluded 2026. All analyses were performed in R 4.6.1 (Supplementary Tables S16 and S17).

## Results

### Study selection and evidence map

WoSCC yielded 863 records, including 862 with cited-reference information, and Scopus yielded 840 records, including 835 with cited references. DOI-based matching with normalized-title fallback linked 727 records across the two databases, leaving 136 WoSCC-specific and 113 Scopus-specific records. Because database coverage was overlapping but non-equivalent, WoSCC and Scopus were analyzed separately.

The PubMed/Europe PMC workflow yielded 843 records and 588 unique articles after deduplication. Screening identified 526 eligible articles, including 481 original studies and 45 reviews. Of 162 locally available full-text records, 15 were excluded from translation-relevant assessment, leaving 147 original studies, corresponding to 30.6% of the eligible original-study corpus (Figure 1; Supplementary Table S15). The full-text subset differed from the remaining 334 original studies in publication year and architecture composition. Mean publication year was 2022.76 versus 2022.07 (standardized mean difference = 0.237), and the overall architecture difference was modest (Cramér’s V = 0.185). Biomacromolecular systems accounted for 8.2% versus 3.0%, pure-drug systems for 9.5% versus 4.5%, and covalent assemblies for 32.7% versus 41.6% in the full-text and remaining subsets, respectively (Supplementary Tables S7 and S8). The full-text subset was therefore availability-selected rather than compositionally identical to the full original-study corpus. Architecture-specific standardized differences ranged from −0.184 to 0.247. The largest absolute differences were observed for biomacromolecular assemblies (0.247), pure-drug assemblies (0.211), and covalent assemblies (−0.184).

**FIGURE 1:**
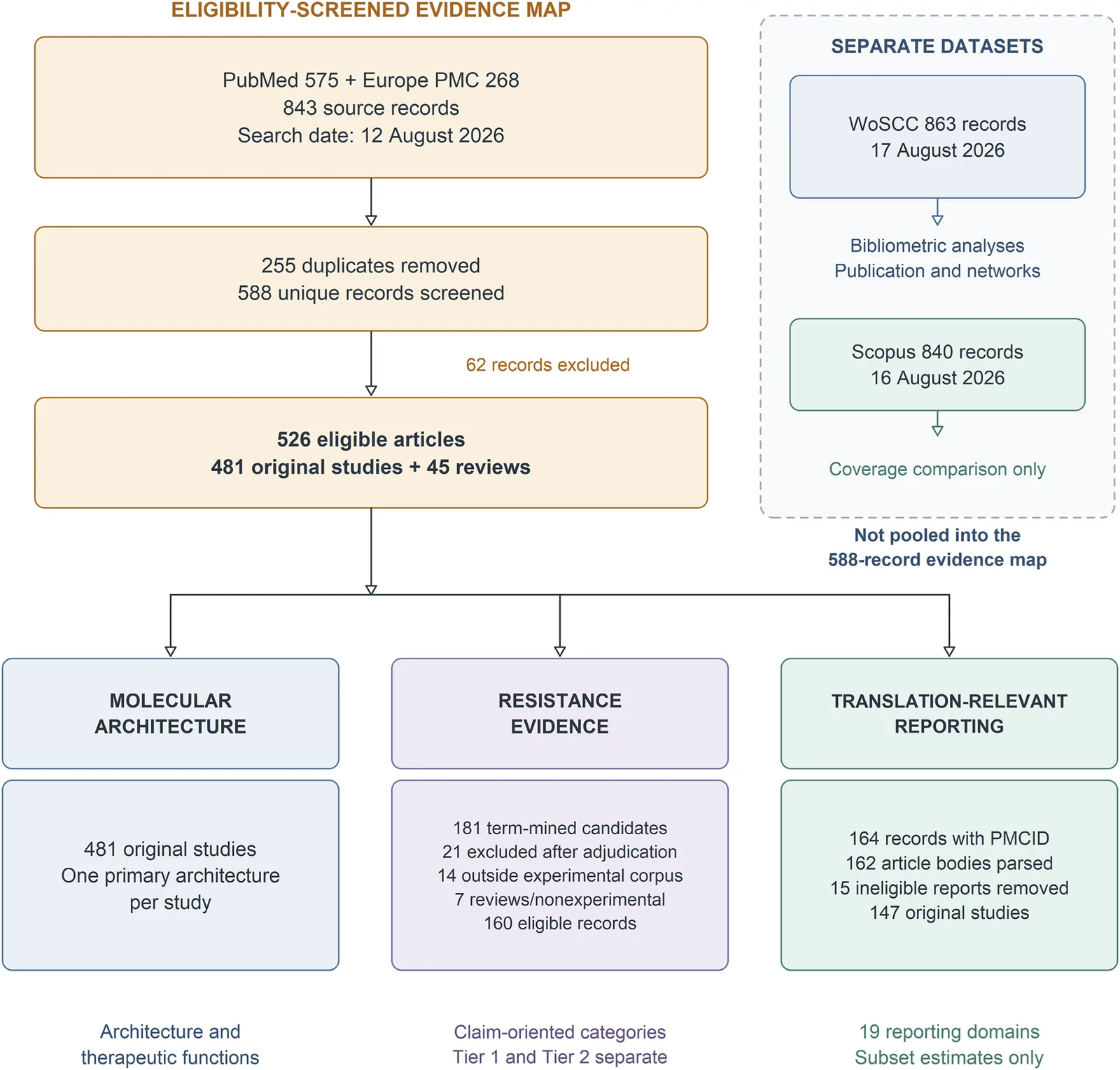
Study selection and analytical framework. Flow diagram showing literature identification, screening, and allocation of records to bibliometric and article-level evidence analyses. WoSCC was used for bibliometric analysis, Scopus for database-coverage assessment, and the screened evidence corpus for molecular-architecture classification, resistance-evidence assessment, and translation-relevant reporting. Neither the WoSCC nor Scopus records were merged into the 588-record evidence map.

### Publication trends, research themes, and molecular architectures

WoSCC publication output reached 140 records in 2025, with a further 96 records indexed by 17 August 2026 (Figure 2). Bibliographic coupling identified 716 visible edges and three communities (modularity = 0.166), while the reference co-citation network contained 761 edges and three communities (modularity = 0.184) (Figures 3, 4). The main knowledge structures reflected early pure-drug and amphiphilic prodrug systems, drug-drug co-assemblies, and multifunctional carrier-free platforms integrating responsive delivery, imaging, phototherapy, and immune modulation. Scopus showed a similar overall growth pattern but different database coverage.

**FIGURE 2:**
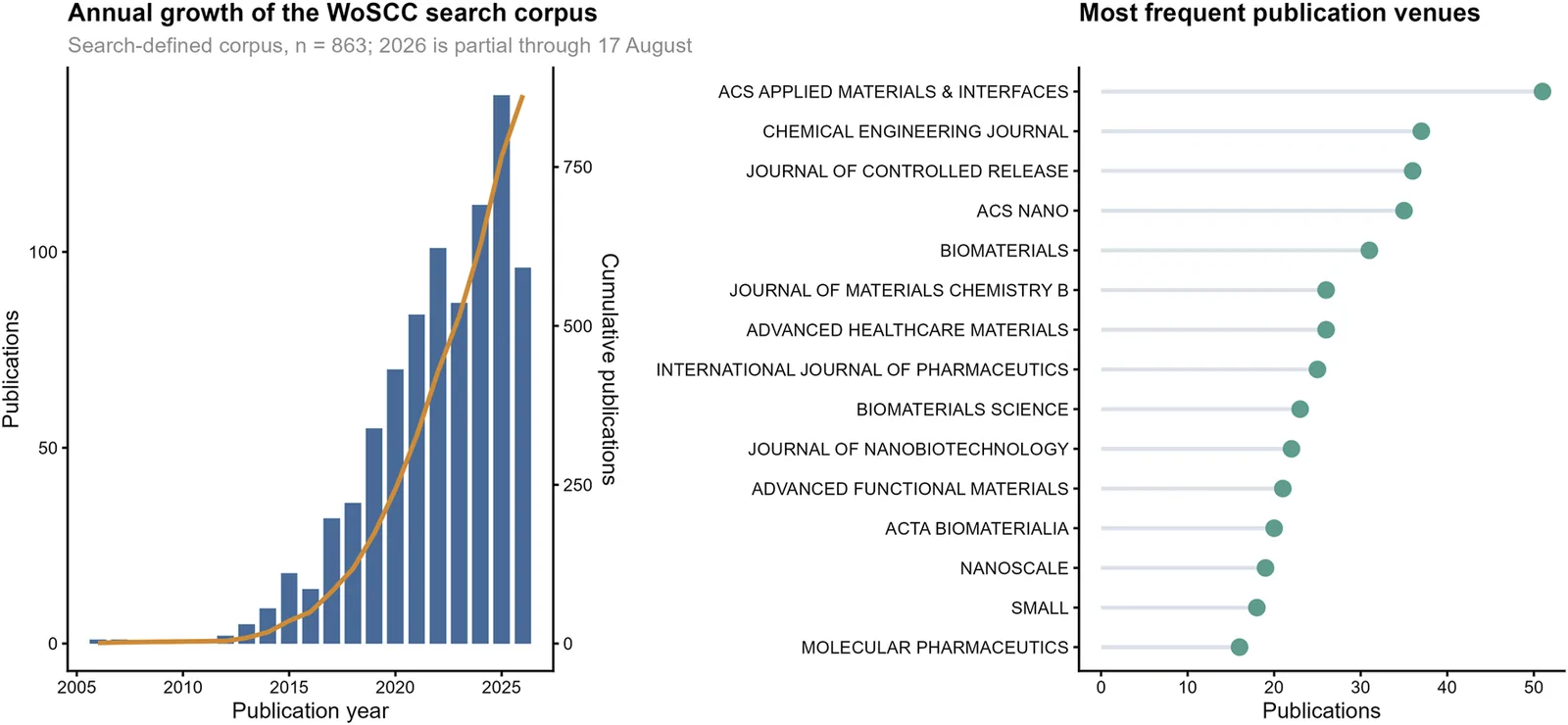
Publication trends and leading journals in carrier-free anticancer nanodrug research. **(a)** Annual and cumulative publication output in the WoSCC corpus. The 2026 count includes records indexed up to 17 August 2026. **(b)** The 15 journals with the highest publication output.

**FIGURE 3:**
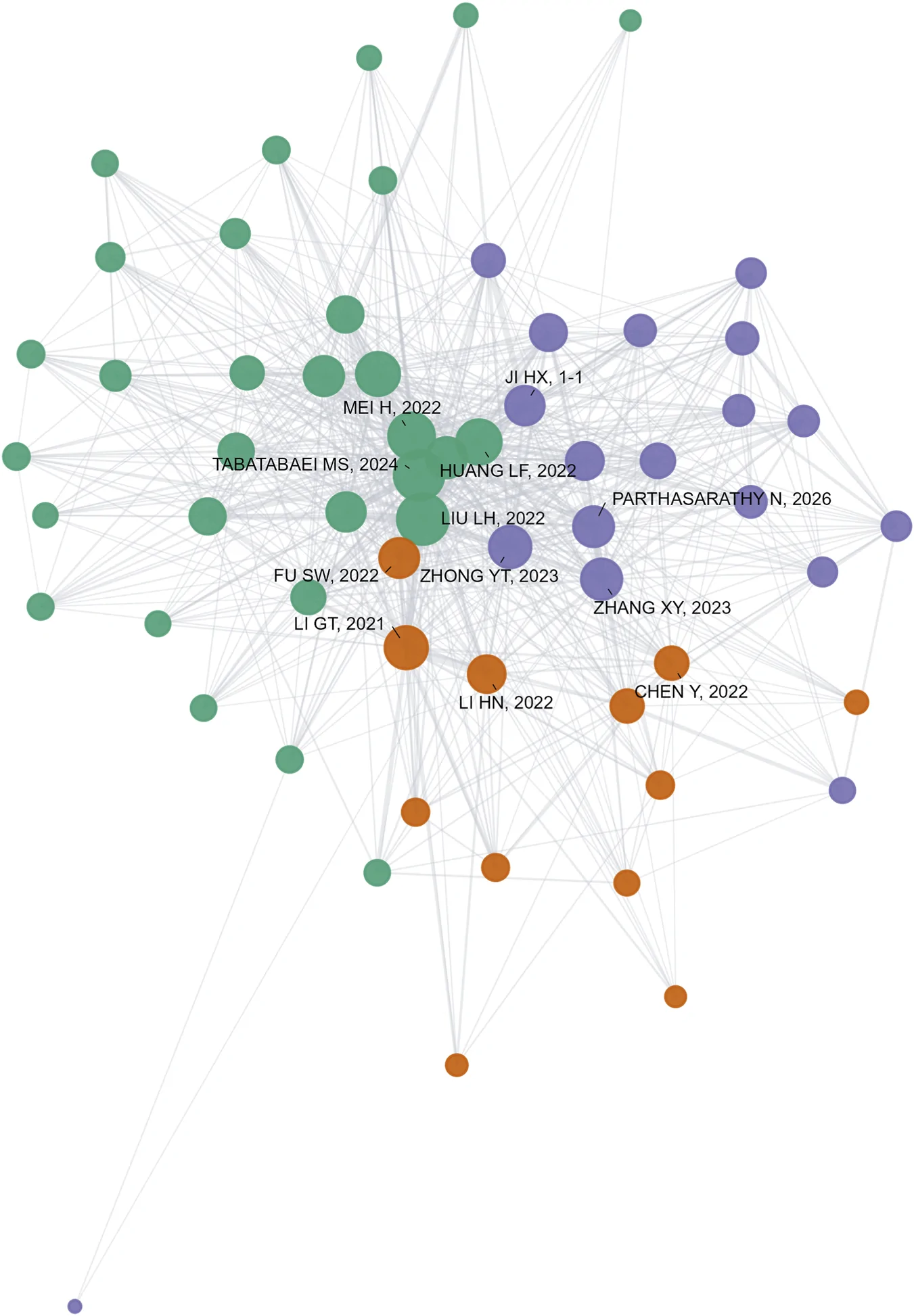
Bibliographic coupling network of carrier-free anticancer nanodrug research. Network of the most strongly coupled documents in the WoSCC corpus. Node size represents weighted coupling strength, links represent shared references, and colors indicate network communities.

**FIGURE 4:**
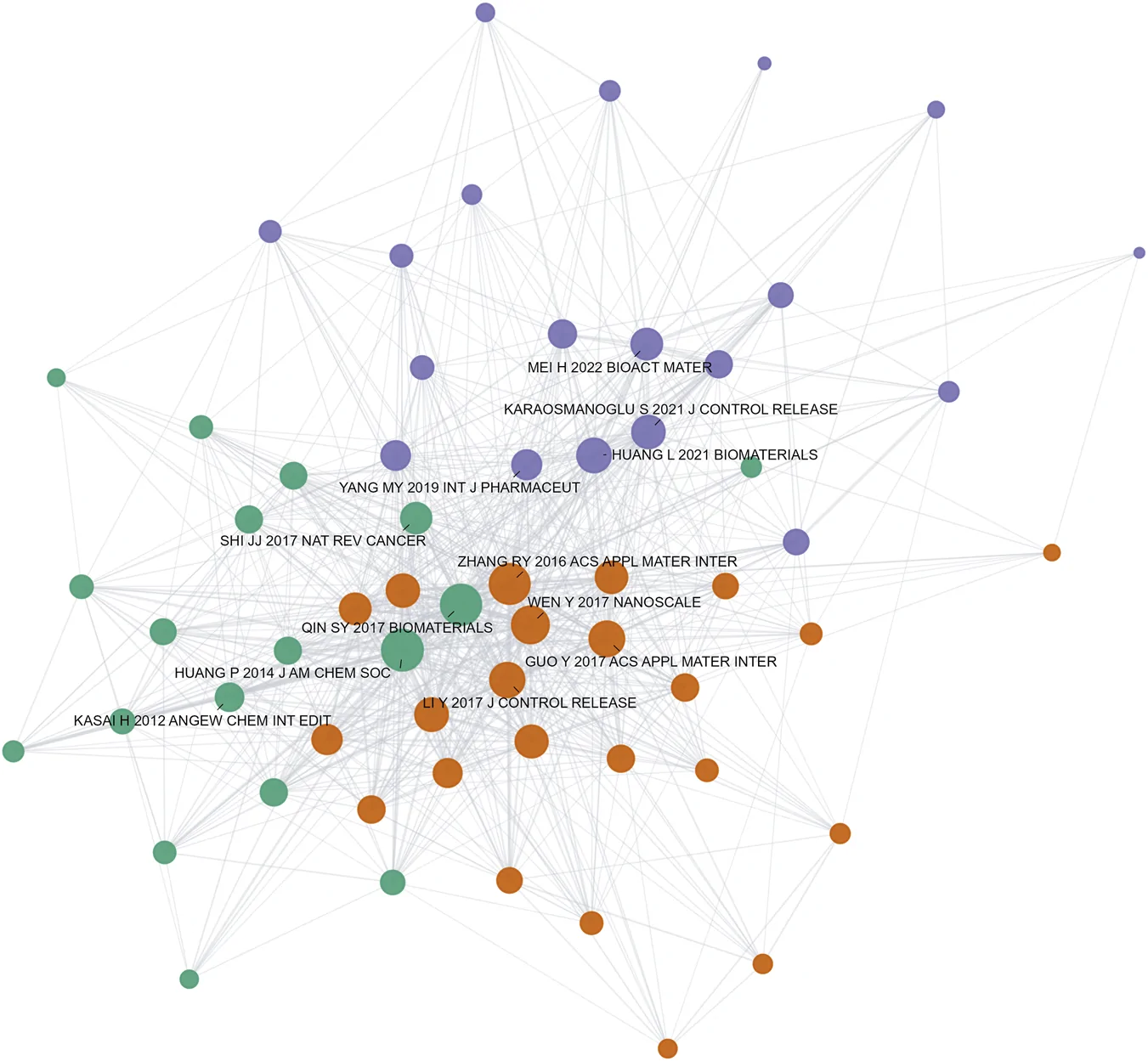
Reference co-citation network of carrier-free anticancer nanodrug research. Co-citation network of the most strongly connected references in the WoSCC corpus. Node size represents weighted co-citation strength, links represent co-citation relationships, and colors indicate network communities.

Keyword analysis showed a shift from broad formulation concepts toward tumor- and mechanism-focused themes (Figure 5). Self-assembly, carrier-free design, drug delivery, photodynamic therapy, and nanomedicine remained prominent, whereas immunotherapy, immunogenic cell death, ferroptosis, and tumor-microenvironment terms became more frequent during 2021–2026. Among the 481 original studies, covalent prodrug/conjugate assemblies were most frequent (n = 187), followed by noncovalent multi-active co-assemblies (n = 182), metal-coordinated systems (n = 60), pure-drug systems (n = 29), and biomacromolecular assemblies (n = 22), with one study unclassified (Figure 6). Therapeutic functions were non-exclusive: chemotherapy was reported in 275 studies, immunotherapy in 156, photodynamic therapy in 143, imaging or image-guided applications in 93, photothermal therapy in 71, regulated cell death in 45, chemodynamic therapy in 25, and gene or RNA therapy in 21. Figure 6 further shows changes in architecture composition over time and the distribution of therapeutic functions across architecture classes.

**FIGURE 5:**
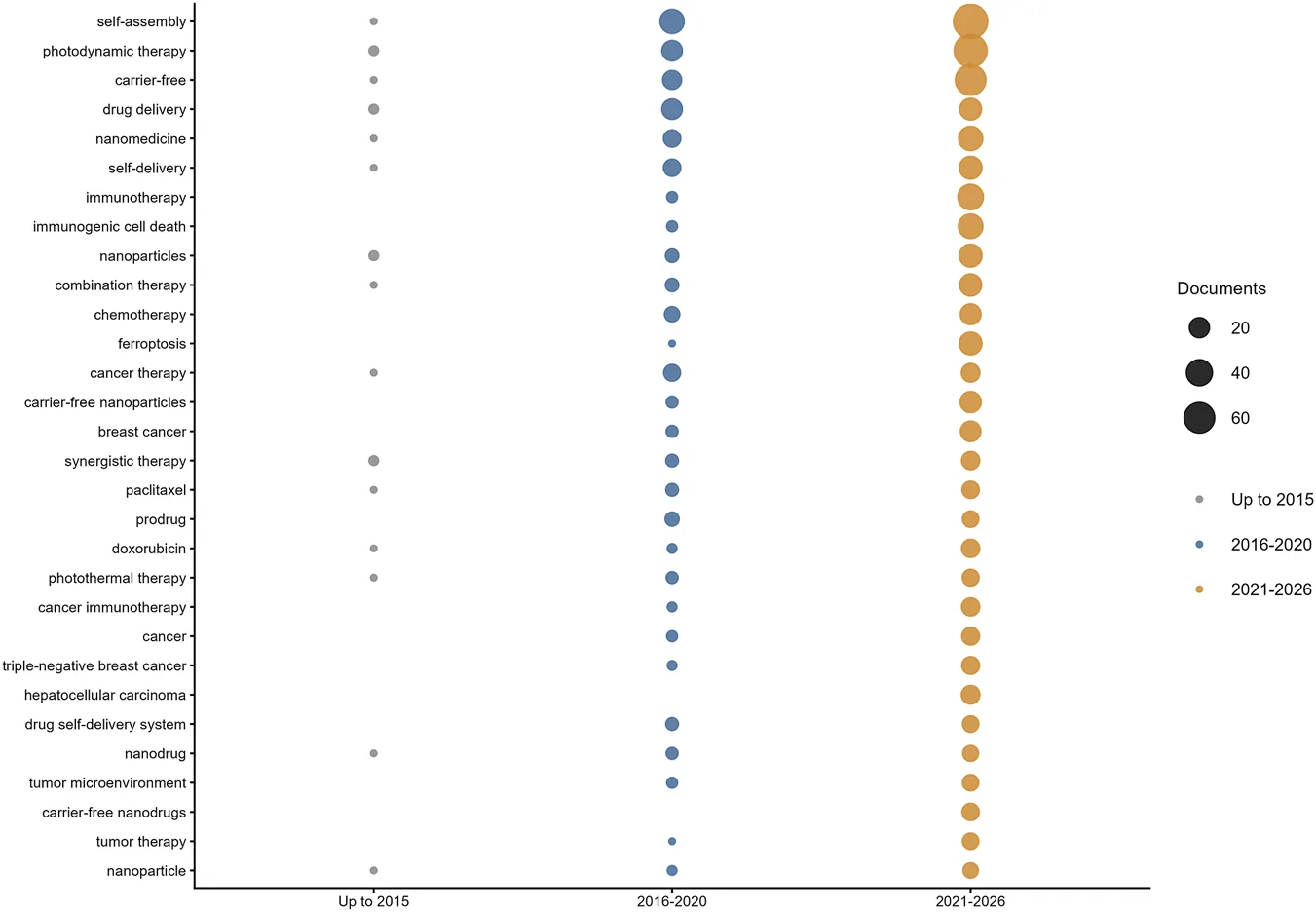
Temporal evolution of research themes based on author keywords. Distribution of the 30 most frequent author keywords across three publication periods: up to 2015, 2016-2020, and 2021-2026. Dot size represents the number of associated documents. The final period includes the incomplete 2026 publication year.

**FIGURE 6:**
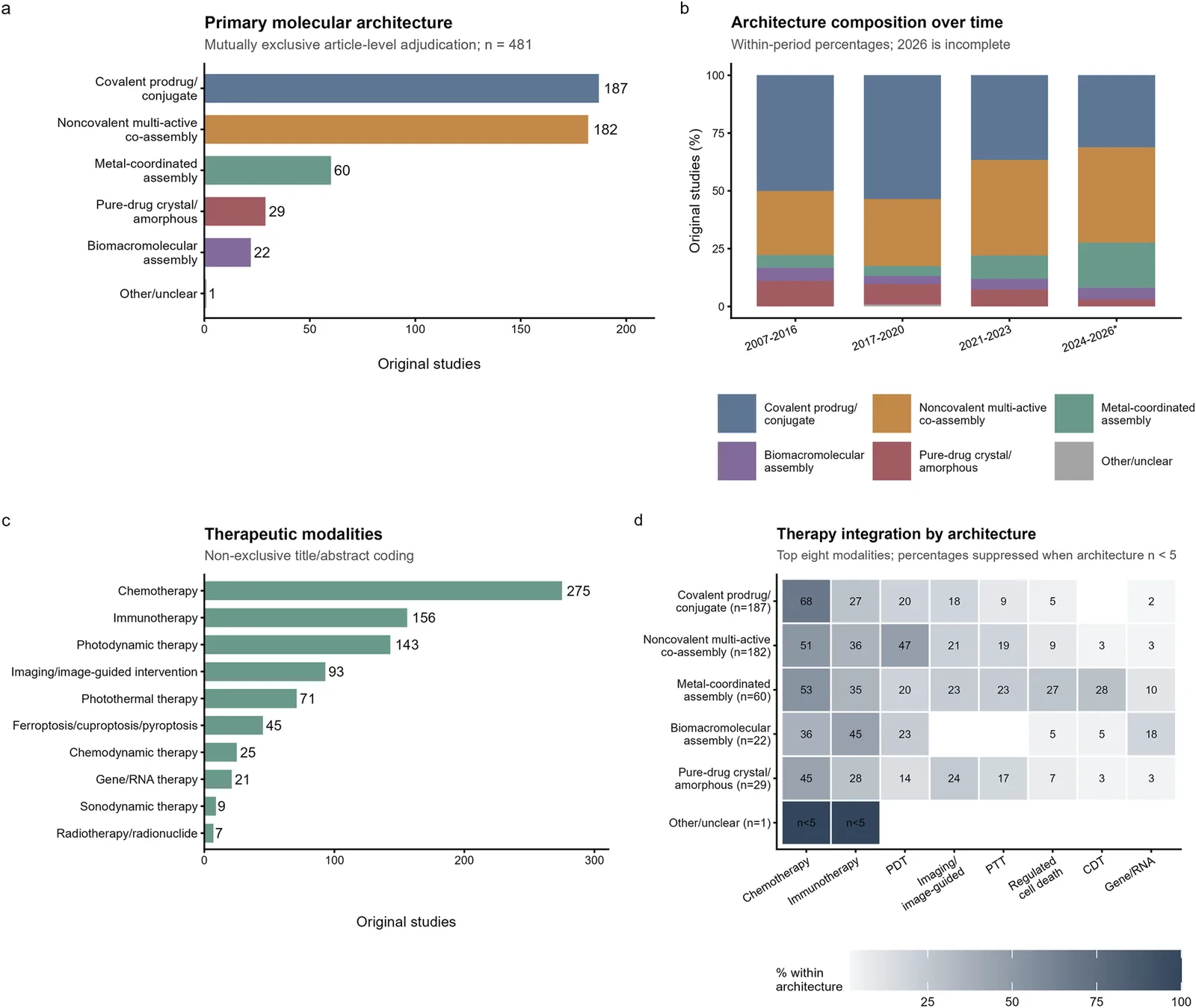
Molecular architectures and therapeutic functions of carrier-free nanodrugs. **(a)** Distribution of primary molecular architectures among 481 original studies. **(b)** Changes in architecture composition across publication periods. **(c)** Distribution of non-exclusive therapeutic modalities. **(d)** Therapeutic modalities across molecular-architecture classes. Primary architecture assignments were mutually exclusive, whereas therapeutic modalities could overlap.

### Resistance evidence and mechanistic landscape

Resistance-related term mining identified 181 candidate records. After exclusion of records outside the experimental corpus (n = 14) and reviews or nonexperimental publications (n = 7), 160 records remained for evidence classification. Among these, 28 studies provided established resistant-model evidence (Tier 1), whereas 15 demonstrated mechanism-aligned sensitization without resistant-model confirmation (Tier 2). Sixty-one studies addressed other treatment-resistance processes, and 56 referred to resistance only as background rationale or interpretation (Figure 7a). Figure 7c distinguishes the evidence sources used for classification; source availability is separate from methodological quality.

**FIGURE 7:**
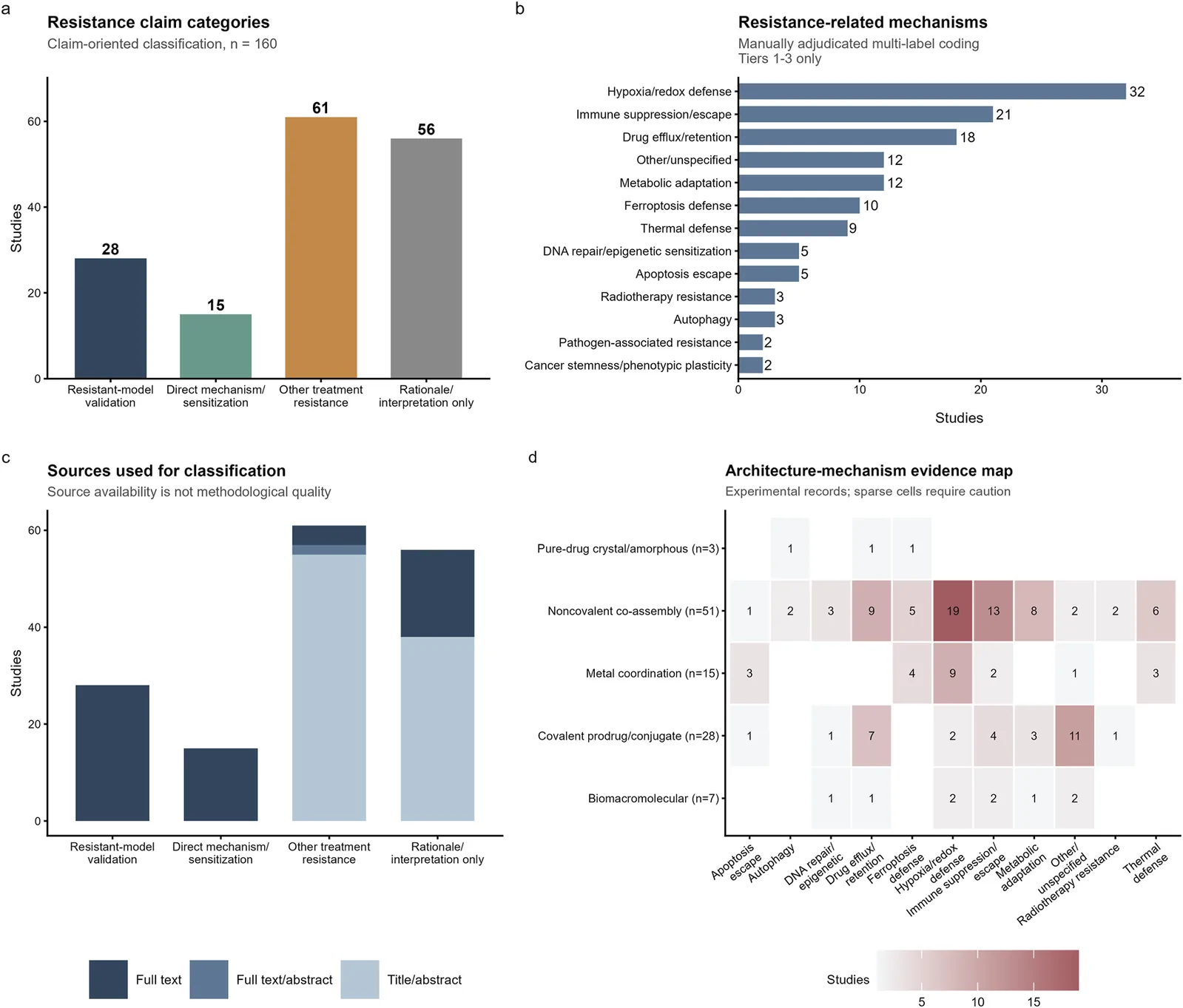
Resistance evidence across mechanisms and molecular architectures. **(a)** Distribution of four claim-oriented resistance categories among 160 eligible records. **(b)** Resistance mechanisms identified among 104 experimental resistance records using multi-label coding. **(c)** Evidence-source patterns across categories. **(d)** Distribution of resistance mechanisms across molecular architectures. Tier one indicates established resistant-model testing and Tier 2 mechanism-aligned sensitization without resistant-model confirmation. Tier 3 denotes other resistance contexts, not inherently weaker methodological quality. Mechanisms are non-exclusive; several architecture-by-mechanism cells contain only one or two studies and should not be interpreted as stable associations.

Tier one studies included cisplatin-, doxorubicin-, paclitaxel-, venetoclax-, irinotecan-, and all-trans retinoic acid-resistant models, together with P-glycoprotein-overexpressing systems. Among 104 experimental resistance records with mechanism coding, hypoxia or redox defense was the most frequent mechanism (n = 32), followed by immune suppression or escape (n = 21), drug efflux or intracellular retention (n = 18), metabolic adaptation (n = 12), ferroptosis defense (n = 10), and thermal defense (n = 9). Mechanism categories were non-exclusive. The corresponding multi-label mechanism frequencies are shown in Figure 7b.

Among the 104 experimentally supported resistance records, noncovalent multi-active co-assemblies were the most common architecture (n = 51), followed by covalent assemblies (n = 28), metal-coordinated assemblies (n = 15), biomacromolecular assemblies (n = 7), and pure-drug systems (n = 3). In the 43 key studies, the architecture-by-Tier 1/Tier 2 association had Cramér’s V = 0.394, an unadjusted Monte Carlo Fisher P = 0.111, and Holm-adjusted P = 0.666. Excluding 2026 left 39 studies and did not provide clearer evidence of an association (V = 0.384; adjusted P = 0.836). Sparse architecture groups limit precision, and these findings do not establish equivalence or comparative therapeutic efficacy. Representative platforms are summarized in Table 2, complete architecture-specific counts and intervals are provided in Supplementary Tables S16 and S17. Supplementary Table S13 provides an expanded set of representative studies. Figure 7d displays architecture-by-mechanism counts.

**TABLE 2:** Representative carrier-free nanodrug platforms with Tier one or Tier 2 resistance evidence. Representative platforms selected to illustrate different molecular architectures, resistance mechanisms, and experimental designs. Tier one indicates validation in an established resistant model with an interpretable comparator, whereas Tier 2 indicates mechanism-aligned sensitization without resistant-model confirmation. Examples are illustrative and not intended to represent all eligible studies.

Evidence tier	Year	Doi	Platform	Primary architecture	Resistance context	Direct readouts	Claim boundary	Selection rationale
Tier 1	2014	10.1002/anie.201309985	Biomimetic RNA-silencing nanocomplex (Wang et al., 2014)	Biomacromolecular active assembly	OVCAR8/ADR multidrug-resistant ovarian cancer	P-glycoprotein silencing and restored drug sensitivity	Resistant-model validation	Early genetically targeted efflux example
Tier 1	2015	10.1021/acsami.5b05347	Dual-anticancer-drug coassembly (Zhao et al., 2015)	Noncovalent multi-active co-assembly	MCF-7R drug-resistant breast cancer	Drug retention P-gp efflux cytotoxicity and colony formation	Resistant-model validation	Direct coassembly-to-efflux evidence chain
Tier 1	2017	10.1016/j.jconrel.2017.09.007	Cisplatin-vorinostat supramolecular nanodrug (Xu et al., 2017)	Covalent prodrug/conjugate assembly	A549/DR cisplatin-resistant tumor	Differential efficacy versus free cisplatin in resistant model	Resistant-model validation	Links molecular integration to epigenetic sensitization
Tier 1	2018	10.1039/c8nr02700e	Celastrol-doxorubicin nanocrystals (Xiao et al., 2018)	Noncovalent multi-active co-assembly	MCF-7/MDR breast cancer	P-gp suppression intracellular accumulation and cytotoxicity	Resistant-model validation	Representative transporter-plus-autophagy design
Tier 1	2021	10.1016/j.ijpharm.2021.120691	Indomethacin-disulfide-paclitaxel nanodrug (Kang et al., 2021)	Covalent prodrug/conjugate assembly	A549/taxol paclitaxel-resistant lung cancer	MRP1 downregulation intracellular delivery and MDR reversal	Resistant-model validation	Named resistant line and transporter endpoint
Tier 1	2023	10.1186/s12951-023-02157-x	Carrier-free supramolecular nano-twin-drug (Yuan et al., 2023)	Noncovalent multi-active co-assembly	HCT8/V irinotecan-resistant colorectal cancer	Resistant cells and resistant xenograft efficacy	Resistant-model validation	Recent *in-vitro*/*in-vivo* resistant-model example
Tier 1	2025	10.1016/j.jconrel.2025.01.050	HO-1 inhibitor-Pt (IV) self-assembled nanomedicine (Liu et al., 2025)	Covalent prodrug/conjugate assembly	A549cisR cisplatin-resistant lung cancer	MDR-related proteins chemosensitization and resistant-cell efficacy	Resistant-model validation	Links stress signaling MDR proteins and chemo-immunotherapy
Tier 1	2025	10.1016/j.biomaterials.2024.122788	Carrier-free resistant-tumor-antigen nanovaccine (Pan et al., 2025)	Biomacromolecular active assembly	Chemotherapy-resistant tumors	Resistant-tumor antigen vaccination and immunotherapy response	Resistant-model validation but immune mechanism distinct from classical efflux MDR	Represents immune-resistance branch without conflating mechanisms
Tier 2	2017	10.1039/c7tb02562a	Aptamer-functionalized ursolic-acid/doxorubicin system (Jiang et al., 2017)	Noncovalent multi-active co-assembly	HER2-overexpressing cancer without established resistant comparator	Intracellular doxorubicin retention and sensitization	Mechanism-aligned sensitization only	Clear efflux/retention readout without Tier 1 model
Tier 2	2019	10.1007/s40820-019-0310-0	Drug nanorod delivering microRNA-101 (Xin et al., 2019)	Pure-drug crystal/amorphous assembly	Chemotherapy self-sensitization without established resistant comparator	Autophagy inhibition and enhanced chemotherapy response	Mechanism-aligned sensitization only	Shows autophagy-mediated sensitization
Tier 1	2020	10.1002/anie.202006895	Precise drug-chemogene conjugates (Zhu et al., 2020a)	Covalent prodrug/conjugate assembly	HeLa/PTX resistant cells and tumors	P-glycoprotein knockdown and active-drug release	Resistant-model validation	Established resistant-model evidence; risk-of-bias domains appraised separately
Tier 2	2022	10.1016/j.biomaterials.2022.121598	Photothermal-differentiation-chemotherapy nanodrug (Xie et al., 2022)	Covalent prodrug/conjugate assembly	Chemoresistant breast cancer stem cells without matched resistant line	Stem-cell differentiation and increased chemotherapy sensitivity	Mechanism-aligned sensitization only	Represents phenotypic-plasticity mechanism
Tier 1	2024	10.1016/j.jconrel.2024.04.007	mRNA-responsive two-in-one nanodrug (Liu et al., 2024)	Noncovalent multi-active co-assembly	MCF-7/ADR resistant cells and tumors	P-glycoprotein mRNA downregulation and reduced MDR phenotype	Resistant-model validation	Established resistant-model evidence; risk-of-bias domains appraised separately
Tier 2	2025	10.1016/j.actbio.2025.02.013	Pathogen-responsive multifunctional nanodrug (Li et al., 2025)	Covalent prodrug/conjugate assembly	*Fusobacterium* nucleatum-associated colorectal chemoresistance	Bacterial elimination oxaliplatin sensitization and immune remodeling	Mechanism-aligned sensitization only	Extends resistance beyond tumor-cell-intrinsic MDR
Tier 2	2026	10.1002/adma.202505951	Carrier-free nano-epidrug (Qi et al., 2026)	Noncovalent multi-active co-assembly	Osteosarcoma chemosensitization without established resistant comparator	m7G tRNA modification histone acetylation DNA damage and doxorubicin sensitivity	Mechanism-aligned sensitization only	Recent epigenetic sensitization design

### Translation-relevant reporting and methodological appraisal

The 43 Tier 1/2 studies underwent methodological appraisal. A sensitive or parental comparator was reported in 18 studies, while active-equivalent controls and formulation characterization were reported in all 43. Replication or sample-size information was available in 32 studies and statistical methods in 39. Among 29 studies with *in vivo* experiments, random allocation was reported in 18 and blinding in none (Supplementary Table S14).

Across the 147 full-text studies, composition definition, particle size or morphology, and *in vitro* efficacy were reported in all studies. *In vivo* efficacy was reported in 122 studies, biodistribution and colloidal stability in 101 each, release profiles in 91, organ histology in 87, serum stability in 86, and assembly verification in 84. Lower reporting frequencies were observed for pharmacokinetics (n = 45), clinically relevant models (n = 44), storage stability (n = 42), normal-cell safety (n = 23), human-related evidence (n = 7), long-term toxicity (n = 4), batch consistency (n = 3), and scalability (n = 3) (Figure 8; Supplementary Table S9). Exploratory architecture associations were imprecisely estimated for pharmacokinetics (V = 0.210, adjusted P = 1.000), colloidal stability (V = 0.236, adjusted P = 0.675), serum stability (V = 0.173, adjusted P = 1.000), storage stability (V = 0.205, adjusted P = 1.000), and *in vivo* efficacy reporting (V = 0.194, adjusted P = 1.000). None showed clear evidence of association after multiplicity adjustment (Supplementary Tables S16 and S17).

**FIGURE 8:**
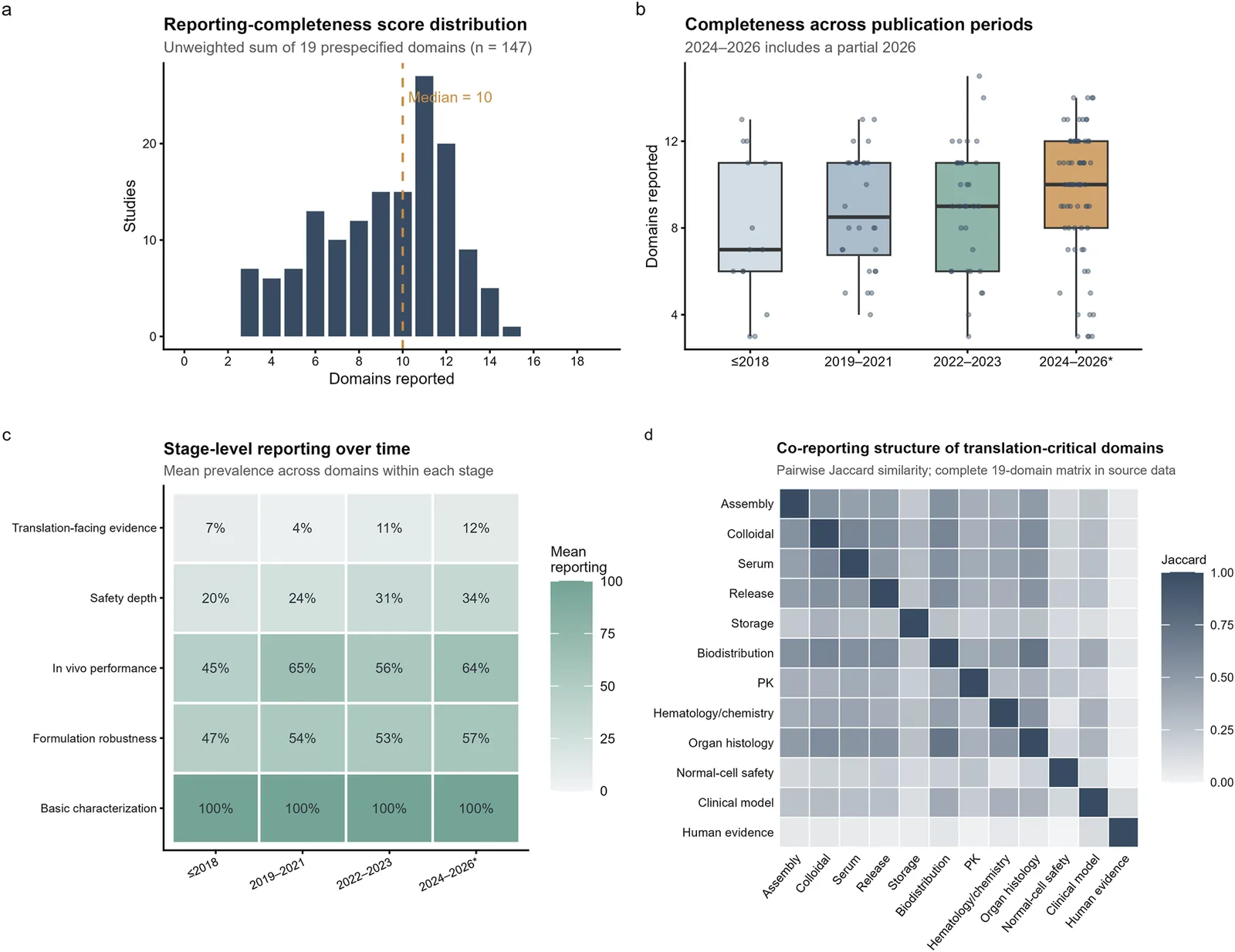
Translation-relevant reporting in carrier-free nanodrug studies. **(a)** Distribution of reporting-completeness scores among 147 full-text original studies. **(b)** Scores across publication periods. **(c)** Unweighted mean reporting frequencies across grouped domains over time. Domains within a group can have markedly different frequencies; group means do not imply equal clinical importance or conceal a required minimum level of evidence. **(d)** Co-reporting patterns based on pairwise Jaccard similarity. Reporting completeness is not formulation quality, efficacy, or readiness. The latest-versus-earliest mean-score difference after excluding 2026 was 1.40 domains (95% CI, −0.49–3.25), compatible with no improvement.

Reporting-completeness scores ranged from 3 to 15 domains, with a median of 10. Median scores increased from 7 in the earliest period to 10 in 2024–2026. The latest-versus-earliest mean difference was 1.87 domains (95% bootstrap CI, 0.00–3.71), but decreased to 1.40 domains (95% CI, −0.49–3.25) after excluding the incomplete 2026 years, providing no clear evidence of sustained temporal improvement. Seven studies met the composite human-related evidence domain (7/147, 4.8%). Only one involved direct clinical administration (1/147, 0.7%), and this concerned fluorescence-guided surgery rather than clinical reversal of drug resistance. Four studies used patient- or donor-derived experimental material (4/147, 2.7%), and two used patient-derived molecular datasets (2/147, 1.4%) (Supplementary Tables S10 and S11). Co-reporting patterns across domains are shown in Figure 8d.

### Reliability of article-level classification

Independent duplicate assessment covered 80 screening decisions, 61 architecture assignments, 40 resistance-category assignments and 20 full-text studies across 19 reporting domains. Pre-consensus agreement was 63/80 for screening (κ = 0.575), 54/61 for architecture (κ = 0.857), 39/40 for resistance categories (unweighted κ = 0.967), and 343/380 study-domain decisions for translation-related reporting (κ = 0.804). These estimates describe the evaluated samples and do not demonstrate complete dual-independent screening of the full corpus.

In a separate full-report assessment of all 43 key Tier one and Tier 2 studies, independent Tier assignments agreed in 43/43 cases (unweighted κ = 1.000). Subsequent source verification corrected two concordant assignments from Tier 2 to Tier 1. These were corrections of shared errors, not inter-reviewer disagreements; complete agreement therefore should not be interpreted as proof of classification accuracy. The final distribution was 28 Tier one and 15 Tier 2 studies. Disagreements elsewhere were resolved jointly, and no third evaluator was used. The distinct reliability exercises and pre-consensus estimates are reported in Supplementary Table S12.

## Discussion

This systematic scoping review and evidence map shows that carrier-free anticancer nanodrugs have expanded in both publication volume and molecular complexity. Covalent and noncovalent multi-active assemblies dominate the experimental literature, while resistant-model validation and development-relevant reporting remain limited in the assessed studies. Resistance-related terminology was considerably more common than validation in established resistant models, while evidence for batch reproducibility, scalability, delayed toxicity, and human relevance remained limited in the available full-text subset. These findings shift the central question from whether a carrier can be removed to whether the resulting architecture can maintain its material properties, support the pharmacological claim being made, and generate evidence required for further development.

### From carrier elimination to molecular integration

Early carrier-free nanomedicines were developed mainly to increase drug loading and enable self-delivery through pure-drug particles or assemblies of active components (Shen et al., 2017; Yang et al., 2019; Karaosmanoglu et al., 2021). More recent systems have expanded toward covalent prodrugs, noncovalent multi-active assemblies, coordination-based systems, and biomacromolecular platforms that combine delivery with imaging, phototherapy, immune modulation, or regulated cell-death pathways (Zhang X. et al., 2023; Ji et al., 2024; Lin et al., 2024). The bibliometric evolution observed here follows the same direction, with increasing attention to tumor-microenvironment regulation, immunogenic cell death, reactive oxygen species, and ferroptosis. These changes illustrate a shift from relatively simple drug-rich nanoparticles toward molecularly integrated systems designed to perform several therapeutic functions simultaneously.

This integration also changes the pharmaceutical properties that need to be controlled. Pure-drug systems remain sensitive to particle state, crystallinity, aggregation, and dissolution, whereas covalent assemblies require control of conjugation efficiency, chemical purity, linker stability, and cleavage behavior (Shim et al., 2019; Fang and Chen, 2024). Noncovalent assemblies depend on intermolecular interactions and can be affected by dilution, serum exposure, and processing conditions (Li et al., 2017; Tang et al., 2025). Coordination-based systems introduce additional dependence on metal-ligand interactions and biological dissociation, while biomacromolecular systems require control of molecular composition and structural stability (Wang et al., 2021). Accessory membranes, targeting ligands, and surface modifications add another layer of biological and colloidal complexity (Singh et al., 2026). Protein-mediated surface engineering outside the carrier-free corpus provides a related example. Panicker et al. (2026) showed that fetal bovine serum engineering altered colloidal behavior and cellular responses of gold nanoparticles, illustrating how surface composition can influence biological performance even when the underlying particle core is unchanged.

Carrier-free status should therefore be understood primarily as a compositional feature rather than as evidence of simplified formulation development. Removing an inactive carrier shifts the source of complexity toward the active components themselves, their molecular interactions, solid-state properties, assembly stability, and release behavior. This distinction is consistent with broader nanomedicine reporting frameworks that emphasize physicochemical characterization and reproducibility rather than particle size or morphology alone (Faria et al., 2018; Leong et al., 2019). It also explains why different carrier-free architectures require different analytical priorities. Solid-state characterization is particularly important for pure-drug particles because polymorphic conversion or reprecipitation can alter dissolution without changing nominal composition. Covalent assemblies require separate measurements of conjugate identity, residual free drug, linker stability, and active-parent release. For noncovalent co-assemblies, the feed ratio should be distinguished from the incorporated ratio and from the ratio retained after dilution or serum exposure (Huang et al., 2014; Shim et al., 2019; Faria et al., 2018; U.S. Food and Drug Administration, 2022).

Related pharmaceutical systems also help define the boundary of the carrier-free concept. Polymeric solid dispersions can improve dissolution and stability but still rely on a carrier matrix (Ramadhan et al., 2026), while core-shell nanoparticles use a structurally distinct shell to protect the active compound or control release (Ramadhan and Ali, 2025). In contrast, the carrier-free systems considered here are characterized by therapeutic or bioactive components forming the principal structural phase. This distinction is important because preparation methods such as nanoprecipitation, crystallization, or microfluidic mixing describe how a particle is produced, but not necessarily the molecular mechanism by which its nanoscale structure is maintained.

### Resistance evidence requires claim-matched models

Drug resistance provided a useful test of whether molecular design was matched by pharmacological evidence. The classification used here separated established resistant-model validation from mechanism-aligned sensitization and from other experimentally studied resistance contexts. Tier one therefore reflects testing in an established resistant phenotype with an interpretable comparator, whereas Tier 2 reflects sensitization or resistance-related mechanism evidence without resistant-model confirmation. Tier 3 covers biologically distinct resistance contexts such as hypoxia-related treatment resistance, thermotolerance, immune escape, and ferroptosis defense. These categories describe the type of claim supported by the experiment rather than a universal hierarchy of study quality.

This distinction is particularly important because cancer resistance is not a single biological state. Drug efflux and intracellular retention are closely related to classical multidrug resistance through their effects on intracellular drug exposure. Carrier-free systems targeting these processes, including RNA-silencing nanocomplexes and co-assembled drug platforms, have restored treatment sensitivity in established resistant models (Wang et al., 2014; Zhao et al., 2015; Xiao et al., 2018; Gao et al., 2020). In contrast, epigenetic regulation, autophagy, metabolism, apoptosis escape, cellular plasticity, hypoxia adaptation, ferroptosis defense, and immune suppression influence treatment response through different mechanisms (Xie et al., 2022; Zhu et al., 2019; Long et al., 2020). Increased intracellular accumulation, transporter modulation, or pathway inhibition may therefore demonstrate a relevant mechanism without demonstrating reversal of an established resistant phenotype.

The appraisal of the 43 Tier one and Tier 2 studies further showed that resistant-model choice alone does not determine the strength of a study. Active-equivalent controls and formulation characterization were well represented, but sensitive or parental comparators, replication information, randomization, and blinding were less consistently reported. A convincing resistance study requires an experimentally defined resistant phenotype, an appropriate comparator, and controls that distinguish the effect of the assembled formulation from differences in dose, exposure, or drug combination. Mechanistic measurements such as transporter activity, intracellular exposure, apoptosis, or pathway regulation then become most informative when they explain an observed change in treatment response.

The exploratory architecture analysis did not identify a clear association with Tier one versus Tier 2 classification. No association with pharmacokinetics, colloidal stability, serum stability, storage stability, or *in vivo* efficacy reporting was clear after multiplicity adjustment. Sparse architecture groups and imprecise estimates limit these comparisons; the results do not establish that architecture is unrelated to evidence generation. Architecture alone should therefore not be used to infer the strength of resistance validation or the completeness of development-related reporting. Interpretation also requires the experimental model, comparator, and endpoints.

### From proof of concept to translation

The translation-relevant analysis revealed a marked imbalance between proof-of-concept efficacy and evidence needed for continued development. Formulation identity, basic particle characterization, and *in vitro* efficacy were widely reported, while pharmacokinetics, storage stability, normal-cell safety, batch consistency, scalability, long-term toxicity, and human evidence were much less frequent. This pattern is consistent with broader experience in nanomedicine, where efficacy studies often advance more rapidly than reproducibility, pharmaceutical characterization, and translation-oriented evaluation (Faria et al., 2018; Anselmo and Mitragotri, 2019; He et al., 2019; Leong et al., 2019; Germain et al., 2020; Shim et al., 2022; Tong et al., 2024).

For carrier-free systems, these gaps are particularly relevant because the assembled structure is itself a major determinant of drug release and exposure. Changes in component purity, molecular ratio, solvent exchange, mixing, purification, or storage can alter assembly state, particle characteristics, and release behavior. Weak-interaction assemblies may be especially sensitive to environmental and processing conditions (Tang et al., 2025), while scale-up places greater demands on critical quality attributes and manufacturing control (Hua et al., 2018; Zhang H. et al., 2023). Drying and storage can further affect aggregation, redispersion, and product stability (Abdelwahed et al., 2006). Reproducible manufacture and storage stability are therefore part of the pharmaceutical performance of the platform rather than issues that arise only after efficacy has been established.

Stability also extends beyond changes in hydrodynamic diameter. A formulation may retain a similar particle size while undergoing chemical degradation, losing one component of a co-assembly, or releasing active drug prematurely. Size distribution, PDI, morphology, surface charge, free-drug fraction, aggregation, serum integrity, and active-species release capture different aspects of this behavior. Dissolution of a solid particle, dissociation of a noncovalent assembly, and cleavage of a prodrug linker also represent different processes and require different analytical interpretation. Manufacturing variables such as concentration, solvent exchange, mixing, purification, residual solvent, drying, and reconstitution can affect these attributes (Crist et al., 2013; Abdelwahed et al., 2006; Zhang H. et al., 2023; U.S. Food and Drug Administration, 2022). Similar challenges involving release control, manufacturing scale-up, and product classification have been described in other bioinspired delivery systems (Singh et al., 2026).

The temporal analysis did not show convincing evidence that these development-related gaps have consistently narrowed. Reporting-completeness scores were higher in recent studies, but after exclusion of the incomplete 2026 publication year the latest-versus-earliest difference was 1.40 domains, with a 95% confidence interval from −0.49 to 3.25. Human evidence remained particularly limited. Seven studies met the composite human-related domain, but only one involved direct clinical administration, representing 0.7% of the full-text subset. That study concerned fluorescence-guided surgery rather than clinical reversal of drug resistance (He et al., 2022). The remaining studies used patient- or donor-derived experimental material or molecular datasets, which provide human relevance but are distinct from direct clinical evaluation of the nanodrug.

## Implications for pharmacological development

The findings suggest that carrier-free nanodrug development requires closer alignment between molecular design, pharmacological claims, and evidence generation. Improved uptake, altered intracellular distribution, prolonged exposure, transporter inhibition, restored apoptosis, and immune modulation represent different biological effects and require different experimental endpoints. Active-equivalent controls and exposure-aware pharmacological evaluation are therefore essential for distinguishing intrinsic formulation effects from changes caused by altered dosing or biodistribution.

A more coherent development path begins with defining the molecular architecture and the material attributes most likely to influence its behavior. Pharmacological testing can then be matched to the intended claim, including resistant models when resistance reversal is proposed. Pharmacokinetic characterization, safety assessment, manufacturing reproducibility, and human relevance become increasingly important as the formulation progresses beyond proof of concept. The six-stage framework in Table 3 and Figure 9 organizes these evidence domains across development, while Table 4 links the main architecture classes with the quality attributes most relevant to their performance. These frameworks are intended to connect formulation design with the evidence required to support pharmacological and translational claims.

**TABLE 3:** Six-stage evidence package for the development of carrier-free anticancer nanodrugs. The framework groups the 19 reporting domains into six development stages. Reporting frequencies refer to the 147-study full-text subset. Stages and domains are unweighted and are intended to organize evidence needs rather than define regulatory or clinical thresholds.

Stage	Required domain	Proposed development priority	Reporting frequency number	Reporting frequency percent	Interpretive role
1 Identity and assembly	Composition defined	Quantify every active and accessory component and its molar or mass ratio	147	100.0	Confirms what the nanodrug is
1 Identity and assembly	Assembly verified	Use orthogonal evidence beyond morphology to support the proposed intermolecular assembly	84	57.1	Links molecular architecture to particle formation
1 Identity and assembly	Size and morphology	Report size distribution dispersity morphology and measurement conditions	147	100.0	Defines critical physical attributes
2 Formulation performance	Colloidal stability	Test time-dependent size or aggregation in relevant media	101	68.7	Tests physical robustness
2 Formulation performance	Serum stability	Assess integrity or aggregation in protein-containing biological medium	86	58.5	Anticipates biological exposure
2 Formulation performance	Release profile	Measure active release under physiologically relevant and trigger-relevant conditions	91	61.9	Connects stimulus response to exposure
2 Formulation performance	Storage stability	Define storage condition duration and retained critical quality attributes	42	28.6	Supports shelf-life development
3 Reproducibility and manufacture	Batch consistency	Report at least three independent preparations with predefined critical quality attributes	3	2.0	Tests reproducibility
3 Reproducibility and manufacture	Scalability	Report scale concentration mixing purification yield and quality attributes after scale change	3	2.0	Tests manufacturability
4 Biological performance	*In vitro* efficacy	Use active-matched free-drug and rational combination controls with exposure-normalized comparisons	147	100.0	Establishes activity without overstating delivery effects
4 Biological performance	Normal-cell safety	Include relevant nonmalignant cells and exposure-matched controls	23	15.6	Defines preliminary selectivity
4 Biological performance	*In vivo* efficacy	Report prespecified tumor-control endpoints against appropriate active-matched controls	122	83.0	Tests organism-level activity
4 Biological performance	Clinically relevant model	Use resistant orthotopic metastatic patient-derived or immunocompetent models when central to the claim	44	29.9	Aligns model with intended claim
5 Exposure and safety	Biodistribution	Quantify organ and tumor exposure with traceable analytical or imaging methods	101	68.7	Connects localization to efficacy and toxicity
5 Exposure and safety	Pharmacokinetics	Report concentration-time exposure and key parameters for active species	45	30.6	Separates formulation effect from dose exposure
5 Exposure and safety	Hematology and biochemistry	Assess systemic laboratory safety using defined timing and reference interpretation	64	43.5	Detects organ-system toxicity
5 Exposure and safety	Organ histology	Assess major organs with stated collection timing and blinded interpretation where feasible	87	59.2	Provides tissue-level safety evidence
5 Exposure and safety	Long-term toxicity	Include delayed observation and recovery when repeat dosing or durable materials justify it	4	2.7	Tests late toxicity
6 Human translation	Human evidence	Report human samples *ex vivo* validation or clinical evidence without equating these levels	7	4.8	Defines the final translational bridge

**FIGURE 9:**
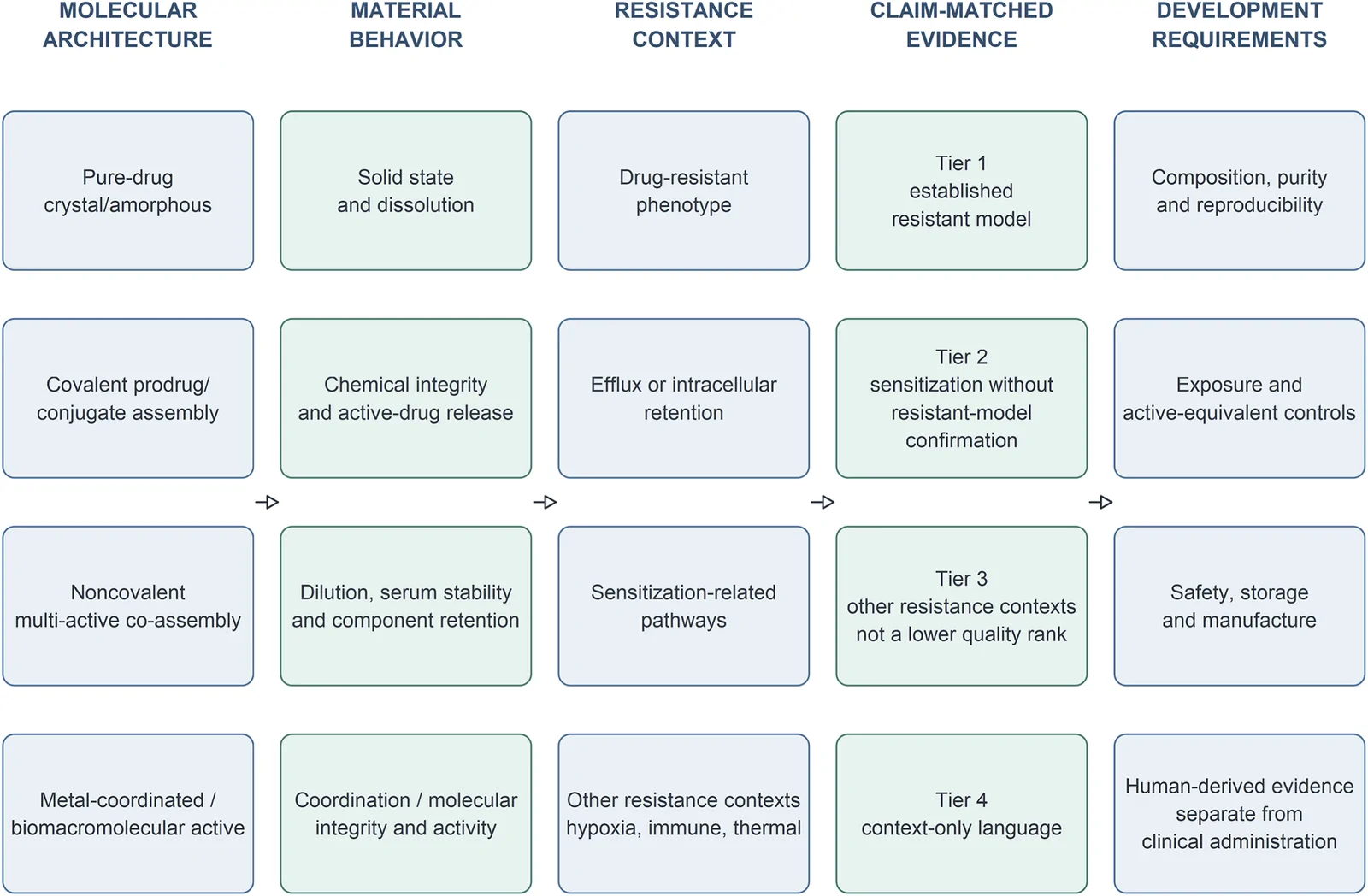
Evidence map linking molecular architecture, resistance evidence, and translational development. Conceptual framework linking molecular architecture, material behavior, resistance-claim interpretation, and development-related evidence. Arrows indicate questions for study design rather than causal relationships established by this review. Tier one and Tier 2 are distinguished by resistant-model confirmation; Tier 3 denotes other resistance contexts and Tier 4 context-only language, not successive levels on a universal quality scale. Reporting estimates apply only to 147 available full-text original studies, not all 481 studies. Development domains are organizing categories, not regulatory weights or validated readiness thresholds. Columns represent complementary design dimensions; horizontal rows do not assign a specific architecture to a resistance category.

**TABLE 4:** Architecture-specific critical quality attributes and characterization priorities. This development framework is informed by the sources cited in the discussion. Suggested measurements depend on formulation and intended use; they are not universal release specifications or newly extracted reporting frequencies.

Architecture	Quality attributes	Complementary measurements	Interpretive boundary
Pure-drug crystal/amorphous particles	Solid form, crystallinity, polymorphism; purity and free drug; dissolution and reprecipitation	PXRD and DSC with microscopy; validated chromatographic assay; dissolution under stated medium and sink conditions	Distinguish dissolution and solid-state conversion from molecular self-assembly
Covalent prodrug/conjugate assemblies	Chemical identity and purity; conjugation stoichiometry; residual free drug; linker stability and cleavage	NMR/MS and chromatography; separate assays for conjugate, parent drug and degradation products	Particle disassembly does not establish active-parent release
Noncovalent multi-active co-assemblies	Incorporated active ratio and loading; unassembled fraction; dilution and serum integrity; component-specific release	Component-resolved assay; perturbation and complementary interaction measurements; separate release profiles	Feed ratio need not equal particle composition or delivered ratio
Metal-coordinated active assemblies	Metal-to-ligand stoichiometry; free metal; coordination state; competitive ligand and pH stability	Elemental and molecular assays; orthogonal coordination evidence; challenge tests in relevant media	Distinguish active coordination assemblies from carrier-dominant frameworks
Biomacromolecular active assemblies	Molecular integrity and heterogeneity; activity; composition, degradation and batch consistency	Identity/purity assays with functional testing; appropriate structural and stability assays	Active core-forming macromolecules differ from inactive protein carriers
All architectures and accessory-modified systems	Size distribution/PDI, morphology, zeta potential under stated conditions, aggregation; chemical/physical storage stability; accessory amount and purity	Complementary sizing and imaging; time-resolved challenge tests; component-specific assay; independent preparation batches	These are development priorities, not a retrospective claim that all properties were measured

### Strengths and limitations

A strength of this study is the combination of field-level bibliometric analysis with article-level assessment of molecular architecture, resistance evidence, and translation-relevant reporting. The different analytical layers retain separate denominators and address different questions, allowing thematic evolution of the field to be considered alongside the experimental evidence reported in individual studies. Independent verification of prespecified samples and separate assessment of the 43 key resistance studies also provided information on the reproducibility of the classification process.

Several limitations remain. WoSCC and Scopus provided overlapping but non-equivalent coverage and were therefore analyzed separately. The bibliometric corpus was broader than the eligibility-screened evidence map, so publication trends and network structure do not directly represent the article-level evidence corpus. Carrier-free terminology also remains inconsistent across the literature. The eligibility framework included drug-dominant crystal or amorphous particles and systems containing accessory materials, and direct evidence of spontaneous molecular self-assembly was not available for every included formulation.

Translation-relevant reporting was assessed in 147 available full-text original studies rather than all 481 original studies. This subset differed from the remaining studies in publication year and architecture composition, so the observed reporting frequencies apply to the audited subset. The Tier one and Tier 2 methodological appraisal was limited to information reported in the publications, and incomplete reporting may not reflect the procedures actually performed. The architecture association analyses were exploratory and involved sparse groups, with no adjustment for factors such as publication year or model choice. Finally, treatment effects were not pooled, therapeutic efficacy was not compared among architecture classes, and the reporting-completeness score was not validated against regulatory or clinical outcomes.

## Conclusion

Carrier-free anticancer nanodrugs encompass diverse molecular architectures and therapeutic functions, while resistance-specific validation and development-relevant evidence remain limited in the assessed literature. Distinguishing resistant-model validation from mechanism-aligned sensitization and other resistance contexts clarifies the scope of resistance claims without treating biologically distinct mechanisms as a single quality hierarchy. Within the available full-text subset, manufacturing reproducibility, long-term toxicity, and direct clinical evaluation remain major evidence gaps.

Further development will depend on closer integration of molecular architecture, material behavior, pharmacological exposure, and claim-matched experimental design. Progress beyond proof-of-concept efficacy will require reproducible manufacture, pharmacokinetic characterization, safety evaluation, and clinically relevant validation. Linking these elements provides a practical framework for strengthening both the pharmacological interpretation and translational development of carrier-free nanodrugs.

## Data Availability

The original contributions presented in the study are included in the article/Supplementary Material, further inquiries can be directed to the corresponding authors.
