# Supplementary material for: Carrier-free self-assembled nanodrugs in cancer therapy: a systematic scoping review and evidence map of molecular architecture, drug resistance, and translation-relevant reporting
Source: Front Pharmacol. 2026 Oct 9;17:1977190. doi: 10.3389/fphar.2026.1977190 (PMC13690816; doi:10.3389/fphar.2026.1977190)
Supplement: Supplementary file 1 [file Table1.docx]

# Supplementary Material

# Supplementary Table S1. Comparison of related bibliometric studies and reviews

Structured comparison of bibliometric studies and reviews relevant to cancer nanomedicine, drug resistance, carrier-free nanodrugs, and nanomedicine translation. Studies were compared by scope, analytical approach, overlap with the present study, and the evidence dimensions not addressed. Within the literature identified by the prespecified search, no study combined a carrier-free or drug-dominant scope with article-level molecular-architecture classification, graded resistance-evidence assessment, and evaluation of translation-relevant reporting.

| **Record type** | **Title** | **Year** | **Journal** | **Doi or pmid** | **Scope** | **Methods** | **Overlap with current study** | **Distinct gap left for current study** | **Verification status** |
| --- | --- | --- | --- | --- | --- | --- | --- | --- | --- |
| Bibliometric | Current perspectives and trend of nanomedicine in cancer: A review and bibliometric analysis | 2023 | Journal of Controlled Release | 10.1016/j.jconrel.2022.10.023 | Broad cancer nanomedicine | WoSCC bibliometrics plus narrative review | Maps growth authors hotspots and broad prodrug/immunotherapy trends | Does not isolate carrier-free systems or adjudicate architecture resistance evidence and reporting domains | DOI and scope verified |
| Bibliometric | Bibliometric and visualized analysis of cancer nanomedicine from 2013 to 2023 | 2024 | Drug Delivery and Translational Research | 10.1007/s13346-023-01485-7 | All antitumor nanocarriers | WoSCC CiteSpace and VOSviewer | Closest broad field-level publication and collaboration map | Corpus is approximately 98980 broad nanocarrier records and lacks carrier-free article-level evidence audit | PMID 38161193 and DOI verified |
| Bibliometric | Current perspectives and trends in nanoparticle drug delivery systems in breast cancer: bibliometric analysis and review | 2023 | Frontiers in Bioengineering and Biotechnology | 10.3389/fbioe.2023.1253048 | Breast-cancer nanoparticle delivery | WoSCC VOSviewer CiteSpace and review | Includes multidrug resistance as one keyword cluster | Disease-specific broad nanoparticles; no resistant-model hierarchy or carrier-free design adjudication | PMID 37771575 PMCID PMC10523396 and DOI verified |
| Bibliometric | Advanced nanoparticles that can target therapy and reverse drug resistance may be the dawn of leukemia treatment: A bibliometrics study | 2022 | Frontiers in Bioengineering and Biotechnology | 10.3389/fbioe.2022.1027868 | Leukemia nanoparticles | WoSCC CiteSpace and VOSviewer | Explicitly discusses targeted therapy and chemotherapy resistance | Disease-specific broad nanoparticles and no article-level resistance evidence grading | PMID 36299285 and DOI verified |
| Bibliometric | Nanomedicine-based cancer immunotherapy: a bibliometric analysis of research progress and prospects | 2024 | Frontiers in Immunology | 10.3389/fimmu.2024.1446532 | Cancer nano-immunotherapy | WoSCC CiteSpace VOSviewer and online platform | Overlaps with the immunotherapy and STING thematic layer | Broad nano-immunotherapy corpus; no carrier-free architecture or development-reporting matrix | PMID 39247199 and DOI verified |
| Bibliometric | Mapping thirty years of tumour-microenvironment-driven drug resistance in breast cancer: a global bibliometric analysis | 2025 | Discover Oncology | 10.1007/s12672-025-03324-2 | Breast-cancer TME-driven resistance | WoSCC Bibliometrix and VOSviewer | Overlaps with nonclassical resistance and TME interpretation | Not nanomedicine-specific and does not distinguish resistant-model evidence from rhetoric | PMID 40772981 and DOI verified |
| Bibliometric | Trends in nanomedicine for colorectal cancer treatment: Bibliometric and visualization analysis (2010-2024) | 2025 | World Journal of Gastrointestinal Oncology | 10.4251/wjgo.v17.i4.102438 | Colorectal-cancer nanomedicine | WoSCC CiteSpace VOSviewer and bibliometrix | Overlaps with disease-level nanomedicine trends | Organ-specific broad nanomedicine and no carrier-free evidence audit | PMCID PMC11995354 and DOI verified |
| Narrative review | Carrier-free nanodrugs for safe and effective cancer treatment | 2021 | Journal of Controlled Release | 10.1016/j.jconrel.2020.10.014 | Carrier-free anticancer nanodrugs | Narrative review | Defines field advantages and design classes | No bibliometric corpus or article-level reporting audit | PMID 33045313 and DOI verified |
| Narrative review | Carrier-free nanodrug: A novel strategy of cancer diagnosis and synergistic therapy | 2019 | International Journal of Pharmaceutics | 10.1016/j.ijpharm.2019.118663 | Carrier-free diagnosis and therapy | Narrative review | Overlaps with assembly strategies and multimodal applications | No bibliometric or evidence-depth analysis | PMID 31493497 and DOI verified |
| Narrative review | Review of Carrier-Free Self-Assembly of Anticancer Nanodrugs | 2023 | ACS Applied Nano Materials | 10.1021/acsanm.3c03315 | Carrier-free supramolecular anticancer nanodrugs | Narrative review | Closest topic-specific design review | No bibliometric landscape resistance hierarchy or reporting-completeness analysis | Publisher DOI and scope verified |
| Narrative review | Advance Progress in Assembly Mechanisms of Carrier-Free Nanodrugs for Cancer Treatment | 2023 | Molecules | 10.3390/molecules28207065 | Carrier-free assembly mechanisms | Narrative review | Overlaps with noncovalent covalent and metal-coordination mechanisms | No field-wide bibliometrics or article-level development audit | PMID 37894544 and DOI verified |
| Narrative review | Research progress in tumor therapy of carrier-free nanodrug | 2024 | Biomedicine & Pharmacotherapy | 10.1016/j.biopha.2024.117258 | Carrier-free tumour therapy | Narrative review | Overlaps with therapeutic modes and future challenges | No systematic bibliometric evidence hierarchy | PMID 39111083 and DOI verified |
| Narrative review | Carrier-Free Nanodrugs: From Bench to Bedside | 2024 | ACS Nano | 10.1021/acsnano.4c09027 | Carrier-free development and translation | Narrative review | Closest translational framing | Does not quantify article-level reporting frequencies or resistance evidence | PMID 39163559 and DOI verified |
| Narrative review | Progress and challenges in the translation of cancer nanomedicines | 2024 | Current Opinion in Biotechnology | 10.1016/j.copbio.2023.103045 | Broad cancer nanomedicine translation | Narrative review | Supports scale manufacturing regulatory and tumour-heterogeneity barriers | Not carrier-free specific and no reporting matrix | PMID 38096768 and DOI verified |
| Narrative review | Survey of Clinical Translation of Cancer Nanomedicines-Lessons Learned from Successes and Failures | 2019 | Accounts of Chemical Research | 10.1021/acs.accounts.9b00228 | Approved and clinical-stage cancer nanomedicines | Clinical landscape review | Supports the preclinical-to-clinical attrition rationale | Not carrier-free specific and focuses clinical products rather than experimental reporting | PMID 31424909 and DOI verified |

# Supplementary Table S2. Eligibility criteria and operational definitions

Inclusion and exclusion criteria used to define the article-level evidence corpus, including publication type, cancer scope, nanoscale state, structural dominance, eligible molecular architectures, and treatment of accessory materials.

| **Domain** | **Inclusion rule** | **Exclusion or boundary rule** |
| --- | --- | --- |
| Publication | English-language journal article or review | Editorial, comment, correction, protocol, conference-only record, preprint without a journal version |
| Disease scope | Cancer diagnosis, treatment, theranostics, or directly relevant tumor model | Non-cancer application without an eligible cancer investigation |
| Nanoscale state | Assembled nanoscale drug-dominant system | Molecular conjugate without an assembled nanoscale state; bulk material only |
| Structural dominance | Active pharmaceutical, bioactive, photosensitizing, sonosensitizing, or immunomodulatory component forms the principal structure | Lipid, polymer, protein carrier, silica, metal-organic framework, or other inactive material forms the dominant structural phase |
| Eligible architectures | Pure-drug crystal/amorphous; covalent prodrug/conjugate; noncovalent multi-active; metal-coordinated active; biomacromolecular active; other drug-dominant assembly | Conventional drug-loaded nanocarrier, even if the abstract uses “self-delivery” or “co-assembly” |
| Accessory materials | Targeting ligands, PEG, hyaluronic acid, stabilizing coatings and membranes may be accessory components when the active core remains structurally dominant. Therapeutically active peptides/proteins may themselves form the core. | Material identity or nominal loading alone does not establish eligibility. An inactive polymer/protein/lipid matrix that carries a loaded drug is excluded; ambiguous composition requires source-level assessment. |
| Reviews | Included in field-level publication and thematic mapping | Excluded from original-study molecular architecture, experimental resistance-mechanism, and translational-readiness denominators |

# Supplementary Table S3. Database search strategies and retrieval results

Database-specific search strategies, search dates, field restrictions, and retrieval results for PubMed, Europe PMC, WoSCC, and Scopus. PubMed and Europe PMC yielded 843 source records and 588 unique records after deduplication, of which 526 met the eligibility criteria for the article-level evidence map. A broader drug-drug co-assembly sensitivity search increased retrieval but also captured conventional carrier-dominant formulations. WoSCC was used as the primary bibliometric corpus and Scopus for database-coverage assessment. Database-specific bibliometric data were analyzed separately.

| **Source** | **Field and filters** | **Final or current query** | **Search date** | **Records retrieved** | **Status** |
| --- | --- | --- | --- | --- | --- |
| PubMed | Title/abstract; journal article or review; English | (((((“carrier-free”[tiab] OR “carrier free”[tiab] OR carrierless[tiab] OR self-deliver*[tiab] OR “self delivery”[tiab] OR “self-delivery”[tiab] OR excipient-free[tiab]) AND (nanodrug*[tiab] OR “nano drug”[tiab] OR nanomedicine*[tiab] OR nanoparticle*[tiab] OR nanoassembl*[tiab] OR nanoprodrug*[tiab] OR nanosystem*[tiab]))) OR “self-assembled nanodrug”[tiab] OR “self assembled nanodrug”[tiab] OR “self-assembled nanomedicine”[tiab] OR “self assembled nanomedicine”[tiab]) AND (cancer*[tiab] OR tumor*[tiab] OR tumour*[tiab] OR neoplasm*[tiab] OR malignan*[tiab] OR carcinoma*[tiab] OR oncolog*[tiab] OR leukemia*[tiab] OR leukaemia*[tiab] OR lymphoma*[tiab]) AND (journal article[pt] OR review[pt]) AND english[la]) | 12 August 2026 | 575 | Executed and sentinel-validated |
| Europe PMC | TITLE_ABS search; English-language and publication-type eligibility applied during screening, not as query filters | (((TITLE_ABS:"carrier-free" OR TITLE_ABS:"carrier free" OR TITLE_ABS:carrierless OR TITLE_ABS:"self-delivering") AND (TITLE_ABS:nanodrug OR TITLE_ABS:nanomedicine OR TITLE_ABS:nanoparticle OR TITLE_ABS:nanoassembly OR TITLE_ABS:nanoprodrug)) OR TITLE_ABS:"self-assembled nanodrug" OR TITLE_ABS:"self assembled nanodrug" OR TITLE_ABS:"self-assembled nanomedicine" OR TITLE_ABS:"self assembled nanomedicine" OR TITLE_ABS:"drug-drug co-assembly" OR TITLE_ABS:"drug drug co-assembly") AND (TITLE_ABS:cancer OR TITLE_ABS:tumor OR TITLE_ABS:tumour OR TITLE_ABS:neoplasm OR TITLE_ABS:carcinoma OR TITLE_ABS:oncology) | 12 August 2026 | 268 | Archived balanced search: 268 retrieved and fetched; 2 of 6 review sentinels recovered. Sentinel testing did not establish exhaustive retrieval. |
| Web of Science Core Collection | Topic; SCI-EXPANDED; Article or Review Article; English; all years through search date | TS=((((“carrier-free” OR “carrier free” OR carrierless OR excipient-free OR self-deliver* OR “self delivery” OR “self-delivery”) NEAR/3 (nanodrug* OR “nano drug*” OR nanomedicine* OR nanoparticle* OR nanoassembl* OR nanoprodrug* OR nanosystem*)) OR “self-assembled nanodrug*” OR “self assembled nanodrug*” OR “self-assembled nanomedicine*” OR “self assembled nanomedicine*”) AND (cancer* OR tumor* OR tumour* OR neoplasm* OR malignan* OR carcinoma* OR oncolog* OR leukemia* OR leukaemia* OR lymphoma*)) | 17 August 2026 | 863 | Complete tagged-text export frozen as ten contiguous chunks; 863 unique accession numbers; cited-reference field available for 862 records and the remaining record reports zero references; primary bibliometric and science-mapping corpus |
| Scopus | TITLE-ABS-KEY; Article or Review; English; all years through search date | TITLE-ABS-KEY(((“carrier-free” OR “carrier free” OR carrierless OR self-deliver* OR “self delivery” OR “self-delivery” OR excipient-free) AND (nanodrug* OR “nano drug” OR nanomedicine* OR nanoparticle* OR nanoassembl* OR nanoprodrug* OR nanosystem*)) OR “self-assembled nanodrug” OR “self assembled nanodrug” OR “self-assembling nanodrug” OR “self assembling nanodrug” OR “self-assembled nanomedicine” OR “self assembled nanomedicine” OR “drug-drug co-assembl*” OR “drug drug co-assembl*”) AND TITLE-ABS-KEY(cancer* OR tumor* OR tumour* OR neoplasm* OR malignan* OR carcinoma* OR oncolog* OR leukemia* OR leukaemia* OR lymphoma*) AND (DOCTYPE(ar) OR DOCTYPE(re)) AND LANGUAGE(english) | 16 August 2026 | 840 | Executed; CSV and RIS each contain 840 records; cited references present for 835 CSV records |

# Supplementary Table S4. Primary molecular-architecture classification

Operational definitions and boundary rules for assigning one mutually exclusive primary molecular architecture according to the mechanism forming the nanoscale core. Accessory ligands, membranes, PEG, and stabilizing coatings did not alter the primary classification when the active core remained structurally dominant. Validation of preliminary rule-assisted classifications showed 74.6% agreement (53/71), below the prespecified 90% threshold, and all 481 original studies were therefore classified by article-level manual adjudication.

| **Architecture** | **Operational definition** | **Common boundary issue** |
| --- | --- | --- |
| Pure-drug crystal/amorphous assembly | One active drug forms the particle through crystallization, amorphous aggregation, or direct pure-drug assembly without a covalent prodrug scaffold | Small surfactant or stabilizer does not automatically make a conventional carrier if it is not the structural phase |
| Covalent prodrug/conjugate assembly | A covalently modified active entity or drug-drug/drug-functional conjugate forms the assembly | Linker and appended active or functional moieties remain part of the defining molecule |
| Noncovalent multi-active co-assembly | Two or more active or bioactive components form the core predominantly through noncovalent interactions | A drug loaded into a preformed inactive carrier is excluded |
| Metal-coordinated active assembly | Metal-ligand coordination involving active or bioactive components defines core formation | Conventional metal-organic frameworks used mainly as carriers are excluded |
| Biomacromolecular active assembly | Therapeutically active peptide, protein, nucleic acid, or related biomacromolecule is a core-forming active component | Inactive albumin or protein carrier systems are excluded |
| Other/unclear drug-dominant assembly | Eligibility is supported but the core-forming mechanism cannot be assigned to the five main classes | Used only after article-level review; not a repository for low-confidence automated labels |

# Supplementary Table S5. Resistance-evidence classification

Operational criteria for four claim-oriented resistance categories. Tier 1 indicates established resistant-model testing, Tier 2 sensitization without resistant-model confirmation, Tier 3 other experimentally studied resistance contexts, and Tier 4 contextual language. Tier 3 is not an inherently weaker level of methodological rigor. Categories do not form a universal ordinal quality scale.

| **Tier** | **Required evidence** | **Permitted interpretation** |
| --- | --- | --- |
| 1 | Established resistant cell line or animal model, or paired sensitive/resistant model with an interpretable comparator | Resistant-model validation; direct resistance evidence |
| 2 | Direct measurement of a resistance mechanism or sensitization effect without an established resistant model | Direct mechanism or sensitization evidence |
| 3 | Experimental evidence for another treatment-resistance process, including hypoxia-related photodynamic resistance, immune escape, thermal defense, or ferroptosis defense | Other treatment resistance; not automatically classical MDR reversal Not intrinsically weaker methodological quality than Tier 1 or Tier 2. |
| 4 | Resistance appears only as background, rationale, general combination advantage, or unsupported interpretation | Context-only language; excluded from mechanism-frequency analyses |

# Supplementary Table S6. Translation-relevant reporting domains and operational criteria

Operational criteria for the 19 binary domains used to assess translation-relevant reporting in the 147-study full-text subset. Automated phrase matching was used to identify candidate evidence, but all classifications required article-level confirmation. Validation of negative assessments prompted targeted full-text review and correction of confirmed false negatives. Detailed reproducibility results are reported in Supplementary Table S12.

| **Stage** | **Domain** | **Minimum evidence required** |
| --- | --- | --- |
| Basic characterization | Composition definition | Identified components plus quantitative composition, feed ratio, loading, stoichiometry, analytical composition, or a structurally confirmed single active entity/prodrug |
| Basic characterization | Particle size/morphology | Direct microscopy, light scattering, or another appropriate sizing or morphology measurement |
| Basic characterization | In vitro efficacy | Direct tumor-cell, spheroid, organoid, or mechanistically linked anticancer endpoint |
| Formulation robustness | Assembly verification | Evidence beyond preparation and morphology, such as perturbation, spectroscopy, thermodynamics, binding, critical association concentration, simulation, or dynamic assembly analysis |
| Formulation robustness | Colloidal stability | Time-resolved or challenge-based size, dispersity, aggregation, sedimentation, or assembly-state assessment |
| Formulation robustness | Serum stability | Direct formulation testing in serum, plasma, whole blood, or a defined serum-containing medium |
| Formulation robustness | Release profile | Quantitative cumulative release, rate, or kinetics at multiple time points or conditions |
| Formulation robustness | Batch consistency | Measured reproducibility across independently prepared batches |
| Formulation robustness | Storage stability | Defined storage or reconstitution condition with a measured formulation endpoint |
| In vivo performance | In vivo efficacy | Measured tumor control, survival, recurrence, metastasis, or therapeutic surgical outcome |
| In vivo performance | Biodistribution | Measured whole-animal, tumor, organ, or tissue distribution after administration |
| In vivo performance | Pharmacokinetics | Systemic concentration-time, half-life, area under the curve, clearance, compartmental, or time-resolved tissue-exposure evidence |
| Safety depth | Normal-cell safety | Direct toxicity or viability endpoint in a nonmalignant cell model |
| Safety depth | Hematology/biochemistry | Measured hematologic or serum-biochemical safety indicators |
| Safety depth | Organ histology | Histological evaluation linked to non-tumor organs |
| Safety depth | Long-term toxicity | Delayed safety assessment or repeated-dose toxicology rather than routine endpoint safety |
| Translation-facing | Scalability | Experimental increase in preparation scale with measured process or product attributes |
| Translation-facing | Clinically relevant model | Patient-derived tissue/organoid, patient-derived xenograft, orthotopic, metastatic, resistant xenograft, or genetically engineered model |
| Translation-facing | Human evidence | Direct clinical application, patient-derived experimental material, or patient-derived molecular evidence, reported separately by evidence type |

# Supplementary Table S7. Comparison of the full-text reporting subset with the remaining original studies

Descriptive comparison of the 147-study full-text subset with the remaining 334 original studies by publication period, molecular architecture, journal, and affiliation country. The comparison was used to assess potential selection associated with full-text availability. Reporting frequencies from the 19-domain assessment therefore apply to the full-text subset and are not interpreted as field-wide prevalence estimates.

| **Variable** | **Level** | **Fulltext number** | **Fulltext percent** | **Other number** | **Other percent** |
| --- | --- | --- | --- | --- | --- |
| Publication period | ≤2018 | 14 | 9.5 | 44 | 13.2 |
| Publication period | 2019-2021 | 28 | 19.0 | 93 | 27.8 |
| Publication period | 2022-2023 | 33 | 22.4 | 70 | 21.0 |
| Publication period | 2024-2026 | 72 | 49.0 | 127 | 38.0 |
| Primary architecture | Biomacromolecular active assembly | 12 | 8.2 | 10 | 3.0 |
| Primary architecture | Covalent prodrug/conjugate assembly | 48 | 32.7 | 139 | 41.6 |
| Primary architecture | Metal-coordinated active assembly | 22 | 15.0 | 38 | 11.4 |
| Primary architecture | Noncovalent multi-active co-assembly | 50 | 34.0 | 132 | 39.5 |
| Primary architecture | Other/unclear drug-dominant assembly | 1 | 0.7 | 0 | 0.0 |
| Primary architecture | Pure-drug crystal/amorphous assembly | 14 | 9.5 | 15 | 4.5 |
| Leading journals | ACS applied materials & interfaces | 3 | 2.0 | 40 | 12.0 |
| Leading journals | ACS nano | 2 | 1.4 | 23 | 6.9 |
| Leading journals | Journal of controlled release : official journal of the Controlled Release Society | 1 | 0.7 | 24 | 7.2 |
| Leading journals | Biomaterials | 1 | 0.7 | 23 | 6.9 |
| Leading journals | Journal of materials chemistry. B | 3 | 2.0 | 17 | 5.1 |
| Leading journals | Advanced healthcare materials | 2 | 1.4 | 17 | 5.1 |
| Leading journals | Journal of nanobiotechnology | 17 | 11.6 | 0 | 0.0 |
| Leading journals | International journal of pharmaceutics | 1 | 0.7 | 16 | 4.8 |
| Leading journals | Acta biomaterialia | 0 | 0.0 | 16 | 4.8 |
| Leading journals | Biomaterials science | 0 | 0.0 | 16 | 4.8 |
| Leading journals | Small (Weinheim an der Bergstrasse, Germany) | 0 | 0.0 | 14 | 4.2 |
| Leading journals | Molecular pharmaceutics | 1 | 0.7 | 13 | 3.9 |
| Leading affiliation countries (full counting) | CN | 125 | 85.0 | 318 | 95.2 |
| Leading affiliation countries (full counting) | US | 32 | 21.8 | 34 | 10.2 |
| Leading affiliation countries (full counting) | GB | 5 | 3.4 | 8 | 2.4 |
| Leading affiliation countries (full counting) | HK | 2 | 1.4 | 10 | 3.0 |
| Leading affiliation countries (full counting) | KR | 2 | 1.4 | 8 | 2.4 |
| Leading affiliation countries (full counting) | SG | 4 | 2.7 | 6 | 1.8 |
| Leading affiliation countries (full counting) | CZ | 2 | 1.4 | 3 | 0.9 |
| Leading affiliation countries (full counting) | IN | 2 | 1.4 | 3 | 0.9 |
| Leading affiliation countries (full counting) | NZ | 0 | 0.0 | 5 | 1.5 |
| Leading affiliation countries (full counting) | MO | 1 | 0.7 | 4 | 1.2 |
| Leading affiliation countries (full counting) | JP | 2 | 1.4 | 3 | 0.9 |
| Leading affiliation countries (full counting) | MG | 0 | 0.0 | 4 | 1.2 |
| OpenAlex country metadata coverage | PMC full-text matrix | 147 | 100.0 |  |  |
| OpenAlex country metadata coverage | Other original studies |  |  | 334 | 100.0 |

# Supplementary Table S8. Effect-size measures for full-text availability differences

Standardized differences and availability odds ratios comparing molecular-architecture composition in the 147-study full-text subset and the remaining 334 original studies. The overall architecture difference had a descriptive Cramér’s V of 0.185, with a maximum absolute architecture-specific standardized difference of 0.247. A Haldane-Anscombe correction was applied to the sparse other or unclear category. These measures describe availability-related differences and were not used as inferential tests of random sampling.

| **Architecture** | **Fulltext number** | **Fulltext percent** | **Other number** | **Other percent** | **Standardized difference** | **Availability odds ratio** | **Or 95ci low** | **Or 95ci high** |
| --- | --- | --- | --- | --- | --- | --- | --- | --- |
| Biomacromolecular active assembly | 12 | 8.2 | 10 | 3.0 | 0.247 | 2.851 | 1.225 | 6.635 |
| Covalent prodrug/conjugate assembly | 48 | 32.7 | 139 | 41.6 | -0.184 | 0.683 | 0.455 | 1.025 |
| Metal-coordinated active assembly | 22 | 15.0 | 38 | 11.4 | 0.109 | 1.381 | 0.788 | 2.418 |
| Noncovalent multi-active co-assembly | 50 | 34.0 | 132 | 39.5 | -0.114 | 0.792 | 0.528 | 1.186 |
| Other/unclear drug-dominant assembly | 1 | 0.7 | 0 | 0.0 | 0.149 | 6.85 | 0.277 | 169.148 |
| Pure-drug crystal/amorphous assembly | 14 | 9.5 | 15 | 4.5 | 0.211 | 2.239 | 1.064 | 4.711 |

# Supplementary Table S9. Uncertainty estimates for translation-relevant reporting domains

Reporting frequencies and Wilson 95% confidence intervals for the 19 translation-relevant domains in the 147-study full-text subset. Each publication was treated as an independent unit of analysis. The intervals quantify uncertainty within this availability-defined subset and should not be interpreted as field-wide prevalence intervals.

| **Domain** | **Reported number** | **Denominator** | **Percent** | **Wilson 95ci low percent** | **Wilson 95ci high percent** | **Estimand** |
| --- | --- | --- | --- | --- | --- | --- |
| Composition_defined | 147 | 147 | 100.0 | 97.5 | 100.0 | Reporting prevalence within PMC-accessible full-text subset |
| Assembly_verified | 84 | 147 | 57.1 | 49.1 | 64.9 | Reporting prevalence within PMC-accessible full-text subset |
| Size_morphology | 147 | 147 | 100.0 | 97.5 | 100.0 | Reporting prevalence within PMC-accessible full-text subset |
| Colloidal_stability | 101 | 147 | 68.7 | 60.8 | 75.6 | Reporting prevalence within PMC-accessible full-text subset |
| Serum_stability | 86 | 147 | 58.5 | 50.4 | 66.2 | Reporting prevalence within PMC-accessible full-text subset |
| Release_profile | 91 | 147 | 61.9 | 53.8 | 69.4 | Reporting prevalence within PMC-accessible full-text subset |
| Batch_consistency | 3 | 147 | 2.0 | 0.7 | 5.8 | Reporting prevalence within PMC-accessible full-text subset |
| In_vitro_efficacy | 147 | 147 | 100.0 | 97.5 | 100.0 | Reporting prevalence within PMC-accessible full-text subset |
| Normal_cell_safety | 23 | 147 | 15.6 | 10.7 | 22.4 | Reporting prevalence within PMC-accessible full-text subset |
| In_vivo_efficacy | 122 | 147 | 83.0 | 76.1 | 88.2 | Reporting prevalence within PMC-accessible full-text subset |
| Biodistribution | 101 | 147 | 68.7 | 60.8 | 75.6 | Reporting prevalence within PMC-accessible full-text subset |
| Pharmacokinetics | 45 | 147 | 30.6 | 23.7 | 38.5 | Reporting prevalence within PMC-accessible full-text subset |
| Hematology_biochemistry | 64 | 147 | 43.5 | 35.8 | 51.6 | Reporting prevalence within PMC-accessible full-text subset |
| Organ_histology | 87 | 147 | 59.2 | 51.1 | 66.8 | Reporting prevalence within PMC-accessible full-text subset |
| Long_term_toxicity | 4 | 147 | 2.7 | 1.1 | 6.8 | Reporting prevalence within PMC-accessible full-text subset |
| Scalability | 3 | 147 | 2.0 | 0.7 | 5.8 | Reporting prevalence within PMC-accessible full-text subset |
| Storage_stability | 42 | 147 | 28.6 | 21.9 | 36.3 | Reporting prevalence within PMC-accessible full-text subset |
| Clinically_relevant_model | 44 | 147 | 29.9 | 23.1 | 37.8 | Reporting prevalence within PMC-accessible full-text subset |
| Human_evidence | 7 | 147 | 4.8 | 2.3 | 9.5 | Reporting prevalence within PMC-accessible full-text subset |

# Supplementary Table S10. Temporal variation in reporting completeness and sensitivity to the incomplete 2026 publication year

Period-specific reporting-completeness scores with bootstrap 95% confidence intervals based on 10000 nonparametric resamples. Comparisons of the earliest and latest publication periods were repeated after exclusion of the incomplete 2026 publication year to assess sensitivity to period definition.

| **Period** | **Number** | **Mean score** | **Mean bootstrap 95ci low** | **Mean bootstrap 95ci high** | **Median score** | **Median bootstrap 95ci low** | **Median bootstrap 95ci high** | **Iqr low** | **Iqr high** |
| --- | --- | --- | --- | --- | --- | --- | --- | --- | --- |
| ≤2018 | 14 | 7.79 | 6.07 | 9.5 | 7.0 | 6.0 | 11.0 | 6.0 | 11.0 |
| 2019-2021 | 28 | 8.82 | 7.82 | 9.79 | 8.5 | 7.0 | 11.0 | 6.8 | 11.0 |
| 2022-2023 | 33 | 9.0 | 8.03 | 9.97 | 9 | 8.0 | 11.0 | 6 | 11 |
| 2024-2026 | 72 | 9.65 | 8.96 | 10.32 | 10.0 | 9.5 | 11.0 | 8.0 | 12.0 |

# Supplementary Table S11. Human-evidence subtypes

Classification of the seven studies meeting the human-evidence domain according to the type of human relevance reported. One study involved direct clinical application, four used patient- or donor-derived experimental material, and two used patient-derived molecular datasets. These categories distinguish human-derived evidence from direct clinical administration.

| **Record id** | **Pmcid** | **Doi** | **Human evidence subtype** | **Evidence basis** | **Interpretive boundary** |
| --- | --- | --- | --- | --- | --- |
| PUB-0368 | PMC9164554 | 10.1186/s12951-022-01467-w | Direct clinical application | Clinical trial of fluorescence-guided hepatocellular-carcinoma resection using the investigated formulation | One clinical application; not evidence that the broader carrier-free field has reached clinical validation |
| PUB-0051 | PMC13440617 | 10.7150/thno.133735 | Patient-derived experimental material | Ex vivo imaging in freshly excised paired tumor and adjacent tissue from 11 patients with triple-negative breast cancer | Ex vivo specimen study; not in-patient therapeutic administration |
| PUB-0176 | PMC12173478 | 10.34133/research.0735 | Patient-derived experimental material | Treatment response tested in patient-derived bladder-cancer organoids | Organoid experiment; not a clinical treatment study |
| PUB-0506 | PMC6567978 | 10.7150/thno.32867 | Patient-derived experimental material | Target expression assessed in paraffin-embedded oral-tongue squamous-cell carcinoma specimens | Human tissue validation of a target; not administration of the nanodrug to patients |
| PUB-0548 | PMC6676892 | 10.1038/s41467-019-11776-4 | Patient-derived experimental material | Erythrocyte membrane obtained from an expired unit of donor packed red blood cells supplied by a hospital transfusion service | Donor-derived formulation material; not administration of the investigated formulation to patients |
| PUB-0101 | PMC11919400 | 10.1016/j.mtbio.2025.101606 | Patient-derived molecular data | TCGA glioblastoma and GTEx normal-brain transcriptomic data used for target analysis | Secondary molecular-data analysis; not patient-level formulation testing |
| PUB-0020 | PMC12955944 | 10.1002/advs.202521525 | Patient-derived molecular data | GEO single-cell and bulk transcriptomic datasets and TCGA-BRCA data used for mechanism analysis | Secondary molecular-data analysis; not patient-level formulation testing |

# Supplementary Table S12. Independent verification and inter-reviewer agreement across coding layers

Prespecified independent verification covered eligibility screening, molecular architecture, resistance classification, and translation-relevant reporting. Agreement was summarized using exact agreement, raw agreement, and unweighted Cohen’s κ before consensus. The 43 key Tier 1/2 resistance studies were assessed separately; two concordant Tier 2 assignments were subsequently corrected to Tier 1 after source verification. Disagreements were resolved by consensus.

| Coding layer | Independent units | Exact agreement | Raw agreement (%) | Unweighted kappa | Disagreements | Final status |
| --- | --- | --- | --- | --- | --- | --- |
| Eligibility screening | 80 | 63/80 | 78.75 | 0.58 | 17 | No change |
| Molecular architecture | 61 | 54/61 | 88.52 | 0.86 | 7 | No change |
| Resistance tier | 40 | 39/40 | 97.5 | 0.97 | 1 | No change |
| Translation-relevant reporting cells | 380 | 343/380 | 90.26 | 0.80 | 37 | No change |
| Separate key-study resistance classification | 43 | 43/43 | 100 | 1.00 | 0 | Two concordant Tier 2 assignments corrected to Tier 1 during subsequent source verification; not inter-reviewer disagreements |

# Supplementary Table S13. Representative resistance studies by mechanism and evidence tier

Representative Tier 1 and Tier 2 studies across drug efflux or intracellular retention, hypoxia or redox adaptation and ferroptosis defense, and immune suppression or escape. The table summarizes molecular architecture, evidence tier, coded mechanism, article-level evidence, and the corresponding interpretive boundary. Tier 1 represents resistant-model validation, whereas Tier 2 represents mechanism-aligned sensitization without resistant-model confirmation. Examples are illustrative rather than exhaustive.

| **Focus_mechanism** | **Record_id** | **Year** | **Doi** | **Title** | **Evidence_tier** | **Evidence_level** | **Molecular_architecture** | **Coded_mechanism** | **Article_level_evidence** | **Interpretive_boundary** |
| --- | --- | --- | --- | --- | --- | --- | --- | --- | --- | --- |
| Efflux / intracellular retention | PUB-0335 | 2022 | 10.1039/d2bm01478e | Erythrocyte membrane camouflaged siRNA/chemodrug nanoassemblies for cancer combination therapy. | Tier 1: resistant-model validation | Full report | Noncovalent multi-active co-assembly | Drug efflux/retention | Anti-P-glycoprotein siRNA was the resistance-modulating component. | Established resistant-model evidence; does not by itself establish low risk of bias. |
| Efflux / intracellular retention | PUB-0366 | 2022 | 10.1039/d1bm01605a | A self-amplified ROS-responsive chemodrug-inhibitor conjugate for multi-drug resistance tumor therapy. | Tier 1: resistant-model validation | Full report | Covalent prodrug/conjugate assembly | Drug efflux/retention | P-gp modulation was tested in MCF-7/ADR tumors. | Established resistant-model evidence; does not by itself establish low risk of bias. |
| Efflux / intracellular retention | PUB-0374 | 2021 | 10.1016/j.ijpharm.2021.120691 | Van der Waals force-driven indomethacin-ss-paclitaxel nanodrugs for reversing multidrug resistance and enhancing NSCLC therapy. | Tier 1: resistant-model validation | Full report | Covalent prodrug/conjugate assembly | Drug efflux/retention | MRP1 downregulation reversed MDR in paclitaxel-resistant A549/taxol cells. | Established resistant-model evidence; does not by itself establish low risk of bias. |
| Efflux / intracellular retention | PUB-0378 | 2021 | 10.1016/j.actbio.2020.12.052 | Supramolecular nanofibers increase the efficacy of 10-hydroxycamptothecin by enhancing nuclear accumulation and depleting cellular ATP. | Tier 1: resistant-model validation | Full report | Covalent prodrug/conjugate assembly | Drug efflux/retention | Enhanced nuclear accumulation and ATP depletion were linked to activity in resistant cells. | Established resistant-model evidence; does not by itself establish low risk of bias. |
| Efflux / intracellular retention | PUB-0384 | 2021 | 10.1039/d1bm00119a | Self-delivery nanomedicine to overcome drug resistance for synergistic chemotherapy. | Tier 1: resistant-model validation | Full report | Noncovalent multi-active co-assembly | Drug efflux/retention | ATP depletion reduced energy-dependent drug efflux in resistant cells. | Established resistant-model evidence; does not by itself establish low risk of bias. |
| Efflux / intracellular retention | PUB-0524 | 2018 | 10.1039/c8nr02700e | Synergistic combination chemotherapy using carrier-free celastrol and doxorubicin nanocrystals for overcoming drug resistance. | Tier 1: resistant-model validation | Full report | Noncovalent multi-active co-assembly | Drug efflux/retention | P-gp suppression and increased accumulation were demonstrated; autophagy was also experimentally implicated. | Established resistant-model evidence; does not by itself establish low risk of bias. |
| Efflux / intracellular retention | PUB-0581 | 2015 | 10.1021/acsami.5b05347 | Nanodrug Formed by Coassembly of Dual Anticancer Drugs to Inhibit Cancer Cell Drug Resistance. | Tier 1: resistant-model validation | Full report | Noncovalent multi-active co-assembly | Drug efflux/retention | Drug retention and P-glycoprotein efflux were tested in MCF-7R cells. | Established resistant-model evidence; does not by itself establish low risk of bias. |
| Efflux / intracellular retention | PUB-0586 | 2014 | 10.1002/anie.201309985 | Biomimetic RNA-silencing nanocomplexes: overcoming multidrug resistance in cancer cells. | Tier 1: resistant-model validation | Full report | Biomacromolecular active assembly | Drug efflux/retention | P-glycoprotein silencing restored sensitivity in OVCAR8/ADR cells. | Established resistant-model evidence; does not by itself establish low risk of bias. |
| Efflux / intracellular retention | PUB-0012 | 2026 | 10.1021/acs.langmuir.5c06575 | Self-Assembly Regulation, Drug Release Behavior, Anti-Multidrug Resistance of Redox-Responsive Gemcitabine-Quinine Nanoassemblies in Glioblastoma Therapy. | Tier 2: mechanism-aligned sensitization | Full report | Covalent prodrug/conjugate assembly | Drug efflux/retention | P-gp expression and intracellular gemcitabine accumulation were directly measured. | Mechanism-aligned sensitization without established resistant-model confirmation; must not be described as validated MDR reversal. |
| Efflux / intracellular retention | PUB-0082 | 2025 | 10.1021/acs.langmuir.5c01856 | Vitamin-Integrated PROTAC Nanoparticle for Combined Therapy of NSCLC. | Tier 2: mechanism-aligned sensitization | Full report | Noncovalent multi-active co-assembly | Drug efflux/retention | P-glycoprotein was downregulated by the PROTAC nanoparticle. | Mechanism-aligned sensitization without established resistant-model confirmation; must not be described as validated MDR reversal. |
| Efflux / intracellular retention | PUB-0096 | 2025 | 10.1038/s41598-025-24027-2 | Synergistic anticancer effects of carrier-free self-assembled doxorubicin and emodin nanoparticles in non-small cell lung cancer. | Tier 2: mechanism-aligned sensitization | Full report | Noncovalent multi-active co-assembly | Drug efflux/retention | ABCB1, ABCC1, and ABCC5 suppression was linked to increased intracellular drug accumulation. | Mechanism-aligned sensitization without established resistant-model confirmation; must not be described as validated MDR reversal. |
| Efflux / intracellular retention | PUB-0142 | 2025 | 10.1186/s12951-025-03539-z | Carrier-free nano-integrated strategy of phototherapy and chemotherapy for synergetic AKT targeted therapy to inhibit cancer metastasis and recurrence. | Tier 2: mechanism-aligned sensitization | Full report | Noncovalent multi-active co-assembly | Drug efflux/retention | P-glycoprotein expression was directly measured during combination therapy. | Mechanism-aligned sensitization without established resistant-model confirmation; must not be described as validated MDR reversal. |
| Efflux / intracellular retention | PUB-0169 | 2025 | 10.1016/j.jcis.2024.08.100 | A computer-aided, heterodimer-based “triadic” carrier-free drug delivery platform to mitigate multidrug resistance in lung cancer and enhance efficiency. | Tier 1: resistant-model validation | Full report | Covalent prodrug/conjugate assembly | Drug efflux/retention | P-glycoprotein suppression increased intracellular drug accumulation. | Established resistant-model evidence; does not by itself establish low risk of bias. |
| Efflux / intracellular retention | PUB-0177 | 2024 | 10.1016/j.jconrel.2024.04.007 | mRNA-responsive two-in-one nanodrug for enhanced anti-tumor chemo-gene therapy. | Tier 1: resistant-model validation | Full report | Noncovalent multi-active co-assembly | Drug efflux/retention | Antisense DNA downregulated P-glycoprotein mRNA. | Established resistant-model evidence; does not by itself establish low risk of bias. |
| Efflux / intracellular retention | PUB-0319 | 2022 | 10.1039/d2nr02064e | Reverse anti-breast cancer drug resistance effects by a novel two-step assembled nano-celastrol medicine. | Tier 1: resistant-model validation | Full report | Pure-drug crystal/amorphous assembly | Drug efflux/retention | P-gp suppression and reduced drug efflux were directly reported. | Established resistant-model evidence; does not by itself establish low risk of bias. |
| Efflux / intracellular retention | PUB-0320 | 2022 | 10.1016/j.jpha.2021.03.001 | Regulation of drug release performance using mixed doxorubicin-doxorubicin dimer nanoparticles as a pH-triggered drug self-delivery system. | Tier 2: mechanism-aligned sensitization | Full report | Covalent prodrug/conjugate assembly | Drug efflux/retention | Differential cytotoxicity was attributed to inhibition of P-glycoprotein efflux. | Mechanism-aligned sensitization without established resistant-model confirmation; must not be described as validated MDR reversal. |
| Efflux / intracellular retention | PUB-0476 | 2020 | 10.1002/anie.202006895 | Carrier-Free Delivery of Precise Drug-Chemogene Conjugates for Synergistic Treatment of Drug-Resistant Cancer. | Tier 1: resistant-model validation | Full report | Covalent prodrug/conjugate assembly | Drug efflux/retention | The chemogene conjugate knocked down P-glycoprotein. | Established resistant-model evidence; does not by itself establish low risk of bias. |
| Efflux / intracellular retention | PUB-0567 | 2017 | 10.1039/c7tb02562a | A carrier-free dual-drug nanodelivery system functionalized with aptamer specific targeting HER2-overexpressing cancer cells. | Tier 2: mechanism-aligned sensitization | Full report | Noncovalent multi-active co-assembly | Drug efflux/retention | Ursolic acid increased doxorubicin sensitization and intracellular drug retention. | Mechanism-aligned sensitization without established resistant-model confirmation; must not be described as validated MDR reversal. |
| Hypoxia / redox / ferroptosis | PUB-0121 | 2025 | 10.1016/j.jconrel.2024.12.057 | Four-pronged reversal of chemotherapy resistance by mangiferin amphiphile. | Tier 1: resistant-model validation | Full report | Noncovalent multi-active co-assembly | Ferroptosis defense | Ferroptosis induction and CAF-associated IL-6/STAT3 suppression were linked to efficacy in paclitaxel-resistant tumors. | Established resistant-model evidence; does not by itself establish low risk of bias. |
| Hypoxia / redox / ferroptosis | PUB-0176 | 2025 | 10.34133/research.0735 | A Multifunctional Fe-EGCG@RSL3 Nanomedicine Synergizes Ferroptosis Induction and Tumor Microenvironment Remodeling for Enhanced Bladder Cancer Immunotherapy. | Tier 2: mechanism-aligned sensitization | Full report | Metal-coordinated active assembly | Ferroptosis defense | The formulation overcame limited RSL3 responsiveness and enhanced anti-PD1 immune activity. | Mechanism-aligned sensitization without established resistant-model confirmation; must not be described as validated MDR reversal. |
| Hypoxia / redox / ferroptosis | PUB-0234 | 2024 | 10.1016/j.canlet.2024.217102 | Aptamer functionalized hypoxia-potentiating agent and hypoxia-inducible factor inhibitor combined with hypoxia-activated prodrug for enhanced tumor therapy. | Tier 2: mechanism-aligned sensitization | Full report | Noncovalent multi-active co-assembly | Hypoxia/redox defense | HIF1alpha antisense prevented hypoxia-induced resistance and sensitized prodrug chemotherapy. | Mechanism-aligned sensitization without established resistant-model confirmation; must not be described as validated MDR reversal. |
| Immune suppression / escape | PUB-0173 | 2025 | 10.1016/j.biomaterials.2024.122788 | A carrier-free nanovaccine combined with cancer immunotherapy overcomes gemcitabine resistance. | Tier 1: resistant-model validation | Full report | Biomacromolecular active assembly | Immune suppression/escape | A resistant-tumor-antigen nanovaccine used immune activation to treat chemotherapy-resistant tumors. | Established resistant-model evidence; does not by itself establish low risk of bias. |
| Immune suppression / escape | PUB-0064 | 2026 | 10.1021/acsnano.5c17081 | Carrier-Free Nanoassembly Suppresses Phase Separation via Ribosome-Inspired Crowding Control for Enhanced Chemo-Immunotherapy. | Tier 2: mechanism-aligned sensitization | Full report | Noncovalent multi-active co-assembly | Immune suppression/escape | mTOR and phase-separation control repolarized macrophages and reduced resistance-associated expression. | Mechanism-aligned sensitization without established resistant-model confirmation; must not be described as validated MDR reversal. |
| Immune suppression / escape | PUB-0112 | 2025 | 10.1016/j.actbio.2025.02.013 | Multifunctional nanodrug for simultaneously combating chemoresistance and immunosuppression in Fusobacterium nucleatum-associated colorectal cancer. | Tier 2: mechanism-aligned sensitization | Full report | Covalent prodrug/conjugate assembly | Immune suppression/escape | Fusobacterium elimination restored chemosensitivity and immune-cell remodeling alleviated immunosuppression. | Mechanism-aligned sensitization without established resistant-model confirmation; must not be described as validated MDR reversal. |
| Immune suppression / escape | PUB-0176 | 2025 | 10.34133/research.0735 | A Multifunctional Fe-EGCG@RSL3 Nanomedicine Synergizes Ferroptosis Induction and Tumor Microenvironment Remodeling for Enhanced Bladder Cancer Immunotherapy. | Tier 2: mechanism-aligned sensitization | Full report | Metal-coordinated active assembly | Immune suppression/escape | The formulation overcame limited RSL3 responsiveness and enhanced anti-PD1 immune activity. | Mechanism-aligned sensitization without established resistant-model confirmation; must not be described as validated MDR reversal. |

# Supplementary Table S14. Study-level methodological appraisal of the 43 Tier 1 and Tier 2 resistance studies

Design-aware methodological appraisal of the 43 Tier 1 and Tier 2 resistance studies, including resistance-model alignment, comparator reporting, formulation characterization, replication or sample-size reporting, statistical methods, and, where applicable, randomization and blinding. No composite quality score was calculated. “Not reported” indicates insufficient information in the available report and does not indicate that a procedure was not performed. Tier 1 and Tier 2 classifications were retained as separate evidence categories. The complete matrix is provided in Supplementary_Table_S14.xlsx.

# Supplementary Table S15. Study-level excluded records and exclusion reasons

Study-level exclusion log for records removed from the 588-record deduplicated public-source corpus. The table provides the excluded records and the primary reason for exclusion. The complete log is provided in Supplementary_Table_S15.xlsx.

# Supplementary Table S16. Exploratory associations between molecular architecture and evidence-related outcomes

Architecture was examined in relation to Tier 1/Tier 2 classification in 43 key resistance studies and to pharmacokinetics, colloidal stability, serum stability, storage stability, and in vivo efficacy reporting in the 147-study full-text subset. Associations were assessed using Monte Carlo Fisher tests and Cramér’s V, with Holm adjustment for multiple testing. Sensitivity analyses excluded 2026.

| **Sensitivity** | **Endpoint** | **n** | **Cramér’s V** | **p** | **p_Holm** |
| --- | --- | --- | --- | --- | --- |
| All years | Tier 1 versus Tier 2 | 43 | 0.39 | 0.11 | 0.67 |
| All years | Pharmacokinetics | 147 | 0.21 | 0.26 | 1 |
| All years | Colloidal_stability | 147 | 0.24 | 0.14 | 0.68 |
| All years | Serum_stability | 147 | 0.17 | 0.49 | 1 |
| All years | Storage_stability | 147 | 0.21 | 0.30 | 1 |
| All years | In_vivo_efficacy | 147 | 0.19 | 0.37 | 1 |
| Excluding 2026 | Tier 1 versus Tier 2 | 39 | 0.38 | 0.21 | 0.84 |
| Excluding 2026 | Pharmacokinetics | 130 | 0.16 | 0.70 | 1 |
| Excluding 2026 | Colloidal_stability | 130 | 0.25 | 0.13 | 0.80 |
| Excluding 2026 | Serum_stability | 130 | 0.20 | 0.40 | 1 |
| Excluding 2026 | Storage_stability | 130 | 0.20 | 0.42 | 1 |
| Excluding 2026 | In_vivo_efficacy | 130 | 0.24 | 0.13 | 0.80 |

# Supplementary Table S17. Architecture-specific proportions and 95% confidence intervals for resistance and translation-related endpoints

Architecture-specific counts, proportions, and Wilson 95% confidence intervals are reported for Tier 1 classification among Tier 1/2 studies and for pharmacokinetics, colloidal stability, serum stability, storage stability, and in vivo efficacy in the full-text subset. Results are shown for all years and after excluding 2026.

| **Sensitivity** | **Endpoint** | **Architecture** | **n** | **Reported** | **Percent** | **CI_low** | **CI_high** |
| --- | --- | --- | --- | --- | --- | --- | --- |
| All years | Tier 1 among Tier 1 and 2 | Covalent prodrug/conjugate assembly | 20 | 15 | 75.00 | 53.13 | 88.81 |
| All years | Tier 1 among Tier 1 and 2 | Noncovalent multi-active co-assembly | 16 | 8 | 50.00 | 28.00 | 72.00 |
| All years | Tier 1 among Tier 1 and 2 | Biomacromolecular active assembly | 4 | 4 | 100.00 | 51.01 | 100.00 |
| All years | Tier 1 among Tier 1 and 2 | Metal-coordinated active assembly | 1 | 0 | 0.00 | 0.00 | 79.35 |
| All years | Tier 1 among Tier 1 and 2 | Pure-drug crystal/amorphous assembly | 2 | 1 | 50.00 | 9.45 | 90.55 |
| All years | Pharmacokinetics | Metal-coordinated active assembly | 22 | 4 | 18.18 | 7.31 | 38.52 |
| All years | Pharmacokinetics | Covalent prodrug/conjugate assembly | 48 | 17 | 35.42 | 23.43 | 49.56 |
| All years | Pharmacokinetics | Biomacromolecular active assembly | 12 | 6 | 50.00 | 25.38 | 74.62 |
| All years | Pharmacokinetics | Noncovalent multi-active co-assembly | 50 | 16 | 32.00 | 20.76 | 45.81 |
| All years | Pharmacokinetics | Pure-drug crystal/amorphous assembly | 14 | 2 | 14.29 | 4.01 | 39.94 |
| All years | Pharmacokinetics | Other/unclear drug-dominant assembly | 1 | 0 | 0.00 | 0.00 | 79.35 |
| All years | Colloidal_stability | Metal-coordinated active assembly | 22 | 16 | 72.73 | 51.85 | 86.85 |
| All years | Colloidal_stability | Covalent prodrug/conjugate assembly | 48 | 37 | 77.08 | 63.46 | 86.69 |
| All years | Colloidal_stability | Biomacromolecular active assembly | 12 | 6 | 50.00 | 25.38 | 74.62 |
| All years | Colloidal_stability | Noncovalent multi-active co-assembly | 50 | 35 | 70.00 | 56.25 | 80.90 |
| All years | Colloidal_stability | Pure-drug crystal/amorphous assembly | 14 | 7 | 50.00 | 26.80 | 73.20 |
| All years | Colloidal_stability | Other/unclear drug-dominant assembly | 1 | 0 | 0.00 | 0.00 | 79.35 |
| All years | Serum_stability | Metal-coordinated active assembly | 22 | 10 | 45.45 | 26.92 | 65.34 |
| All years | Serum_stability | Covalent prodrug/conjugate assembly | 48 | 32 | 66.67 | 52.54 | 78.32 |
| All years | Serum_stability | Biomacromolecular active assembly | 12 | 6 | 50.00 | 25.38 | 74.62 |
| All years | Serum_stability | Noncovalent multi-active co-assembly | 50 | 30 | 60.00 | 46.18 | 72.39 |
| All years | Serum_stability | Pure-drug crystal/amorphous assembly | 14 | 7 | 50.00 | 26.80 | 73.20 |
| All years | Serum_stability | Other/unclear drug-dominant assembly | 1 | 1 | 100.00 | 20.65 | 100.00 |
| All years | Storage_stability | Metal-coordinated active assembly | 22 | 4 | 18.18 | 7.31 | 38.52 |
| All years | Storage_stability | Covalent prodrug/conjugate assembly | 48 | 16 | 33.33 | 21.68 | 47.46 |
| All years | Storage_stability | Biomacromolecular active assembly | 12 | 1 | 8.33 | 1.49 | 35.39 |
| All years | Storage_stability | Noncovalent multi-active co-assembly | 50 | 18 | 36.00 | 24.14 | 49.86 |
| All years | Storage_stability | Pure-drug crystal/amorphous assembly | 14 | 3 | 21.43 | 7.57 | 47.59 |
| All years | Storage_stability | Other/unclear drug-dominant assembly | 1 | 0 | 0.00 | 0.00 | 79.35 |
| All years | In_vivo_efficacy | Metal-coordinated active assembly | 22 | 21 | 95.45 | 78.20 | 99.19 |
| All years | In_vivo_efficacy | Covalent prodrug/conjugate assembly | 48 | 36 | 75.00 | 61.22 | 85.08 |
| All years | In_vivo_efficacy | Biomacromolecular active assembly | 12 | 11 | 91.67 | 64.61 | 98.51 |
| All years | In_vivo_efficacy | Noncovalent multi-active co-assembly | 50 | 41 | 82.00 | 69.20 | 90.23 |
| All years | In_vivo_efficacy | Pure-drug crystal/amorphous assembly | 14 | 12 | 85.71 | 60.06 | 95.99 |
| All years | In_vivo_efficacy | Other/unclear drug-dominant assembly | 1 | 1 | 100.00 | 20.65 | 100.00 |
| Excluding 2026 | Tier 1 among Tier 1 and 2 | Noncovalent multi-active co-assembly | 13 | 8 | 61.54 | 35.52 | 82.29 |
| Excluding 2026 | Tier 1 among Tier 1 and 2 | Covalent prodrug/conjugate assembly | 19 | 15 | 78.95 | 56.67 | 91.49 |
| Excluding 2026 | Tier 1 among Tier 1 and 2 | Biomacromolecular active assembly | 4 | 4 | 100.00 | 51.01 | 100.00 |
| Excluding 2026 | Tier 1 among Tier 1 and 2 | Metal-coordinated active assembly | 1 | 0 | 0.00 | 0.00 | 79.35 |
| Excluding 2026 | Tier 1 among Tier 1 and 2 | Pure-drug crystal/amorphous assembly | 2 | 1 | 50.00 | 9.45 | 90.55 |
| Excluding 2026 | Pharmacokinetics | Covalent prodrug/conjugate assembly | 43 | 14 | 32.56 | 20.49 | 47.48 |
| Excluding 2026 | Pharmacokinetics | Noncovalent multi-active co-assembly | 44 | 12 | 27.27 | 16.35 | 41.85 |
| Excluding 2026 | Pharmacokinetics | Pure-drug crystal/amorphous assembly | 14 | 2 | 14.29 | 4.01 | 39.94 |
| Excluding 2026 | Pharmacokinetics | Metal-coordinated active assembly | 18 | 4 | 22.22 | 9.00 | 45.21 |
| Excluding 2026 | Pharmacokinetics | Biomacromolecular active assembly | 10 | 4 | 40.00 | 16.82 | 68.73 |
| Excluding 2026 | Pharmacokinetics | Other/unclear drug-dominant assembly | 1 | 0 | 0.00 | 0.00 | 79.35 |
| Excluding 2026 | Colloidal_stability | Covalent prodrug/conjugate assembly | 43 | 32 | 74.42 | 59.76 | 85.07 |
| Excluding 2026 | Colloidal_stability | Noncovalent multi-active co-assembly | 44 | 30 | 68.18 | 53.44 | 80.00 |
| Excluding 2026 | Colloidal_stability | Pure-drug crystal/amorphous assembly | 14 | 7 | 50.00 | 26.80 | 73.20 |
| Excluding 2026 | Colloidal_stability | Metal-coordinated active assembly | 18 | 13 | 72.22 | 49.13 | 87.50 |
| Excluding 2026 | Colloidal_stability | Biomacromolecular active assembly | 10 | 4 | 40.00 | 16.82 | 68.73 |
| Excluding 2026 | Colloidal_stability | Other/unclear drug-dominant assembly | 1 | 0 | 0.00 | 0.00 | 79.35 |
| Excluding 2026 | Serum_stability | Covalent prodrug/conjugate assembly | 43 | 27 | 62.79 | 47.86 | 75.62 |
| Excluding 2026 | Serum_stability | Noncovalent multi-active co-assembly | 44 | 26 | 59.09 | 44.41 | 72.31 |
| Excluding 2026 | Serum_stability | Pure-drug crystal/amorphous assembly | 14 | 7 | 50.00 | 26.80 | 73.20 |
| Excluding 2026 | Serum_stability | Metal-coordinated active assembly | 18 | 7 | 38.89 | 20.31 | 61.38 |
| Excluding 2026 | Serum_stability | Biomacromolecular active assembly | 10 | 4 | 40.00 | 16.82 | 68.73 |
| Excluding 2026 | Serum_stability | Other/unclear drug-dominant assembly | 1 | 1 | 100.00 | 20.65 | 100.00 |
| Excluding 2026 | Storage_stability | Covalent prodrug/conjugate assembly | 43 | 14 | 32.56 | 20.49 | 47.48 |
| Excluding 2026 | Storage_stability | Noncovalent multi-active co-assembly | 44 | 16 | 36.36 | 23.78 | 51.13 |
| Excluding 2026 | Storage_stability | Pure-drug crystal/amorphous assembly | 14 | 3 | 21.43 | 7.57 | 47.59 |
| Excluding 2026 | Storage_stability | Metal-coordinated active assembly | 18 | 3 | 16.67 | 5.84 | 39.22 |
| Excluding 2026 | Storage_stability | Biomacromolecular active assembly | 10 | 1 | 10.00 | 1.79 | 40.42 |
| Excluding 2026 | Storage_stability | Other/unclear drug-dominant assembly | 1 | 0 | 0.00 | 0.00 | 79.35 |
| Excluding 2026 | In_vivo_efficacy | Covalent prodrug/conjugate assembly | 43 | 31 | 72.09 | 57.31 | 83.25 |
| Excluding 2026 | In_vivo_efficacy | Noncovalent multi-active co-assembly | 44 | 35 | 79.55 | 65.50 | 88.85 |
| Excluding 2026 | In_vivo_efficacy | Pure-drug crystal/amorphous assembly | 14 | 12 | 85.71 | 60.06 | 95.99 |
| Excluding 2026 | In_vivo_efficacy | Metal-coordinated active assembly | 18 | 18 | 100.00 | 82.41 | 100.00 |
| Excluding 2026 | In_vivo_efficacy | Biomacromolecular active assembly | 10 | 9 | 90.00 | 59.58 | 98.21 |
| Excluding 2026 | In_vivo_efficacy | Other/unclear drug-dominant assembly | 1 | 1 | 100.00 | 20.65 | 100.00 |
